# Antibiotic use and ileocolonic immune cells in patients receiving fecal microbiota transplantation for refractory intestinal GvHD: a prospective cohort study

**DOI:** 10.1177/20406207211058333

**Published:** 2021-12-21

**Authors:** Walter Spindelboeck, Bettina Halwachs, Nadine Bayer, Bianca Huber-Krassnitzer, Eduard Schulz, Barbara Uhl, Lukas Gaksch, Stefan Hatzl, Victoria Bachmayr, Lisa Kleissl, Patrizia Kump, Alexander Deutsch, Georg Stary, Hildegard Greinix, Gregor Gorkiewicz, Christoph Högenauer, Peter Neumeister

**Affiliations:** Division of Gastroenterology and Hepatology, Department of Internal Medicine, Medical University of Graz, Graz, Austria; Division of Gastroenterology and Hepatology, Department of Internal Medicine, Medical University of Graz, Graz, Austria; Department of Dermatology, Medical University of Vienna, Vienna, Austria; Division of Hematology, Department of Internal Medicine, Medical University of Graz, Graz, Austria; Division of Hematology, Department of Internal Medicine, Medical University of Graz, Graz, Austria; Division of Hematology, Department of Internal Medicine, Medical University of Graz, Graz, Austria; Division of Hematology, Department of Internal Medicine, Medical University of Graz, Graz, Austria; Division of Hematology, Department of Internal Medicine, Medical University of Graz, Graz, Austria; Department of Dermatology, Medical University of Vienna, Vienna, Austria; Department of Dermatology, Medical University of Vienna, Vienna, Austria; Ludwig Boltzmann Institute for Rare and Undiagnosed Diseases, Vienna, Austria; Division of Gastroenterology and Hepatology, Department of Internal Medicine, Medical University of Graz, Graz, Austria; Division of Hematology, Department of Internal Medicine, Medical University of Graz, Graz, Austria; Department of Dermatology, Medical University of Vienna, Vienna, Austria; Ludwig Boltzmann Institute for Rare and Undiagnosed Diseases, Vienna, Austria; CeMM Research Center for Molecular Medicine of the Austrian Academy of Sciences, Vienna, Austria; Division of Hematology, Department of Internal Medicine, Medical University of Graz, Graz, Austria; Institute of Pathology, Medical University of Graz, Graz, Austria; Division of Gastroenterology and Hepatology, Department of Internal Medicine, Medical University of Graz, Auenbruggerplatz 15, 8036 Graz, Austria; BioTechMed-Graz, Graz, Austria; Division of Hematology, Department of Internal Medicine, Medical University of Graz, Graz, Austria

**Keywords:** antibiotics, fecal microbiota transplantation, gastrointestinal graft-*versus*-host disease, regulatory T cells, T cells, type 3 innate lymphoid cells

## Abstract

**Introduction:**

Treatment-refractory, acute graft-*versus*-host disease (GvHD) of the lower gastrointestinal tract (GI) after allogeneic hematopoietic stem cell transplantation is life threatening and lacks effective treatment options. While fecal microbiota transplantation (FMT) was shown to ameliorate GI-GvHD, its mechanisms of action and the factors influencing the treatment response in humans remain unclear.

The objective of this study is to assess response to FMT treatment, factors influencing response, and to study the mucosal immune cell composition in treatment-refractory GI-GvHD.

**Methods:**

Consecutive patients with treatment-refractory GI-GvHD were treated with up to six endoscopically applied FMTs.

**Results:**

We observed the response to FMT in four out of nine patients with severe, treatment refractory GI-GvHD, associated with a significant survival benefit (*p* = 0.017). The concomitant use of broad-spectrum antibiotics was the main factor associated with FMT failure (*p* = 0.048). In addition, antibiotic administration hindered the establishment of donor microbiota after FMT. Unlike in non-responders, the microbiota characteristics (e.g. α- and β-diversity, abundance of anaerobe butyrate-producers) in responders were more significantly similar to those of FMT donors. During active refractory GI-GvHD, an increased infiltrate of T cells, mainly Th17 and CD8^+^ T cells, was observed in the ileocolonic mucosa of patients, while the number of immunomodulatory cells such as regulatory T-cells and type 3 innate lymphoid cells decreased. After FMT, a change in immune cell patterns was induced, depending on the clinical response.

**Conclusion:**

This study increases the knowledge about the crucial effects of antibiotics in patients given FMT for treatment refractory GI-GvHD and defines the characteristic alterations of ileocolonic mucosal immune cells in this setting.

## Introduction

Acute gastrointestinal graft-*versus*-host disease (GI-GvHD), a serious complication of allogeneic hematopoietic stem cell transplantation (allo-HSCT), is characterized by the destruction of the host intestinal epithelia by donor- and resident-host-derived immune cells.^1–3^ Despite steroid treatment, around 40–65% of patients develop refractory disease which is associated with high mortality.^4,5^ Recently, the Janus-kinase 1/2 inhibitor ruxolitinib proved superior to the best-available care for steroid-refractory acute GvHD.^6^ However, effective salvage therapies for treatment-refractory GI-GvHD are urgently needed.^7^

Recent evidence points toward the host–microbiome interplay as a key modulator of GI-GvHD development.^8^ Severe intestinal dysbiosis, a disruption of the gut microbiome diversity and composition, seems to be associated with an increased risk of developing GI-GvHD and with survival after allo-HSCT.^9–11^ However, the consequences of intestinal dysbiosis on disease-driving or disease-modulating immune cells have yet to be elucidated.

Fecal microbiota transplantation (FMT) attenuates intestinal dysbiosis and has been used to ameliorate steroid-refractory or steroid-dependent GI-GvHD in several small studies.^12–19^ Although FMT protocols differ, the results were promising in achieving an improvement in bacterial diversity, associated to sustained clinical responses in a notable proportion of patients and a survival benefit in responders.^14–16^ However, it is still unclear which factors impact on the success of FMT in patients with GI-GvHD.

Although animal studies have provided evidence that FMT may promote protective immune signals within the innate immune system, no human studies have yet been conducted to explore the cellular components of the intestinal immune system in treatment-refractory GI-GvHD.^20,21^

Treatment with preemptive and therapeutic, concomitant, broad-spectrum antibiotics is frequently indicated in GI-GvHD patients. However, none of the previous studies which are inconsistent regarding concomitant antibiotic use have specifically addressed the influence of these antibiotics on FMT outcome.

We herein report a prospective single-center study on repeated colonoscopic FMTs in 10 consecutive patients with severe and treatment-refractory GI-GvHD and elucidate the factors associated with the response to FMT therapy in our cohort.

## Materials and methods

### Study design and patients

We evaluated consecutive patients with GI-GvHD (grading according to the modified Glucksberg^22^ criteria, Przepiorka *et al.*^23^) after allo-HSCT after refractoriness to methylprednisolone 2 mg/kg body weight per day (i.e. either progression of GI-GvHD after 3 days of steroid administration, or no change after 7 days, or no cessation after 14 days of steroids) was confirmed. Then, patient stool was tested by 16S rRNA analysis, and patients were offered colonoscopic FMT when a loss of the physiologic intestinal flora, as assessed by a pathologist with expertise in microbiota analyses, combined with a highly reduced bacterial richness (assessed semi-quantitatively) was evident.

Patients provided their written informed consent during a compassionate-use program between September 2014 and January 2017 (patients A–C; some descriptive data were published in Spindelboeck et al.^13^), and a prospective clinical cohort study was conducted between February 2017 and December 2019 [patients D–J; local institutional review board (IRB) number 29-027 ex 16/17, clinicaltrials.gov-identifier: NCT03819803]. All patients who received FMT for GI-GvHD at our center were followed until death or censoring on 31 December 2019 and are included in this article.

### Outcome measures

The primary endpoint was sustained remission [complete response (CR) or partial response (PR)] of GI-GvHD 90 days after commencement of first FMT. CR was defined as stool volumes <500 ml and absence of GI symptoms. PR was defined as decrease in the GI-GvHD stage ⩾1. Patients with a minimal survival of 14 days after the first FMT were included in the response analysis. Secondary endpoints were overall and GI-GvHD-related survival as well as recurrence of GI-GvHD. Safety endpoints included severe adverse events (SAEs) and suspected unexpected serious adverse reactions occurring up to 48 h after FMT.

### Colonoscopy and FMT

FMTs consisted of an ileo-colonoscopy and administration of 200 ml tested and suspended donor feces into the right colon or the terminal ileum as it is the standard at our center for colonoscopic FMT. Weekly FMT administrations were scheduled and up to six FMTs were performed per patient.

Endoscopies were performed with standard equipment (Olympus, Hamburg, Germany) in sedated patients by gastroenterologists. Forceps biopsies were performed to document the course of the disease histologically and to monitor the immune cell infiltrate by immunofluorescence staining.

### Donor selection, stool preparation, and testing

FMT donors were either healthy relatives of the patients or healthy volunteers. After the fourth patient, we exclusively used banked and frozen (–70°C) stool portions for FMTs due to easier disposability. All but one stool donor were different from the respective stem cell donor.

Potential stool donors were examined according to international FMT guidelines^24^ for chronic and infectious diseases. Details are provided in the Supplementary Methods.

### Microbiota analyses

Raw read data (19,106 839; mean: 91,247 ± 87,593; minimum: 4404; maximum: 797,367; number of samples: 208) were analyzed using QIIME2 (https://qiime2.org,version2019.7), according to online manuals (https://docs.qiime2.org/2019.10/tutorials/moving-pictures/, accessed December 2019). Details are provided in the Supplementary Methods. 16SrRNA gene amplicon datasets analyzed during this study are deposited and available *via* the European Nucleotide Archive (ENA, https://www.ebi.ac.uk/ena) under accession number PRJEB39834 (http://www.ebi.ac.uk/ena/data/view/PRJEB39834).

### Multicolor immunofluorescence

Cryo- and paraffin sections were prepared from intestinal biopsies of GvHD-affected sites and controls (persons without gastrointestinal pathologies that had undergone screening colonoscopy) and stained for T-cell subtypes and type 3 innate lymphoid cells (ILC3; antibodies listed in Supplementary Table 4). Protocols for immunofluorescence labeling of cells on acetone-fixed cryosections were adapted from Brüggen *et al.*^25^ Details are provided in the Supplementary Methods.

### Statistics

The *p* values less than 0.05 were considered as statistically significant if not stated otherwise. Survival analyses were made by Kaplan–Meier estimates and compared by log-rank test. Discriminatory cutoffs for antibiotic use regarding clinical response were calculated using receiver operating characteristics and Youden’s index for the percentage of days without antibiotic therapy after commencement of FMTs and the duration of the longest period without antibiotics (all IBM SPSS Statistics 26, IBM Corp., Armonk, NY, USA). Patients were excluded from a particular analysis when the respective data were missing.

Statistical significance of α- and β-diversity indices between groups (baseline, donors, responders, and non-responders) was calculated using QIIME2 (diversity α-group significance: Kruskal–Wallis test; diversity β-group significance, general and pairwise: PERMANOVA and 999 permutations, at a rarefication depth of 5672). The *p* values for multiple comparisons have been adjusted by the False Discovery Rate. Discriminatory feature analysis between groups, at genus level, was performed by LEfSe (Linear discriminant analysis Effect Size) using the all-against-all multiclass comparison strategy. Proportions of microbiota transferred from donor to post-FMT samples were predicted using the R implementation of SourceTracker (version 1.0.1).

## Results

### Study population

From September 2014 to December 2019, we evaluated 26 and included 10 patients with treatment-refractory GI-GvHD (see Supplementary Figure 1). For the condensed baseline patient characteristics before FMTs and the concomitant medications during FMTs, see Table 1. The detailed clinical characteristics (Supplementary Table 1) and medications (Supplementary Figure 2) are reported in the supplement.

**Table 1.** Patient baseline characteristics upon first FMT and concomitant medications during FMT treatment.

		All patients	Responders	Other patients	*p*
		(*n* = 10)	(*n* = 4)	(*n* = 6)	
Baseline characteristics					
Age	Median (range)	54 (24–67)	57 (42–67)	53 (24–61)	0.476
Sex	Female (%)	5 (50)	2 (50)	3 (50)	1.000
Onset of lower GI-GvHD after allo-HSCT in days	Median (range)	30 (11–465)	68 (11–465)	30 (18–122)	0.914
Lines of GvHD therapies before first FMT	Median (range)	4 (2–6)	4 (3–6)	5 (2–6)	0.825
Methylprednisolone dose upon first FMT (mg)	Median (range)	149 (48–180)	160 (84–168)	145 (48–180)	0.390
Methylprednisolone dose upon first FMT (mg/kg)	Median (range)	1.8 (0.8–2)	1.9 (1.5–2)	1.8 (0.8–2)	0.590
Stool volume per day (maximum in liters)	Median (range)	3.8 (1.7–7.8)	5.1 (3.0–7.8)	3.4 (1.7–5.4)	0.352
Severity of GI-GvHD upon first FMT	Median (range)	4 (2–4)	4 (2–4)	4 (3–4)	0.761
Severity of overall GvHD upon first FMT	Median (range)	IV (IV)	IV (IV)	IV (IV)	1.000
Concomitant medications during FMTs					
Long-term broad-spectrum antibiotics^a^	Yes (%)	5 (100)	0 (0)	5 (83)	0.048
Intravenous methylprednisolone	Yes (%)	10 (100)	4 (100)	6 (100)	1.000
Budesonide	Yes (%)	4 (40)	1 (25)	3 (50)	0.571
Calcineurin inhibitor (cyclosporine, tacrolimus)	Yes (%)	6 (60)	1 (25)	5 (83)	0.190
Etanercept	Yes (%)	8 (80)	4 (100)	4 (67)	0.467
Ruxolitinib	Yes (%)	4 (40)	0 (0)	4 (67)	0.076
Vedolizumab	Yes (%)	4 (40)	1 (25)	3 (50)	0.571
Extracorporeal photopheresis	Yes (%)	4 (40)	2 (50)	2 (33)	1.000
Lines of GvHD therapies concomitantly to FMTs	Median (range)	5 (3–7)	4 (3–5)	6 (3–7)	0.067

allo-HSCT, allogeneic hematopoietic stem cell transplantation; FMT, fecal microbiota transplantation; GI-GvHD, gastrointestinal graft-*versus*-host disease.

Mann–Whitney test, Fisher’s exact test.

a Yes – broad-spectrum antibiotics were given in parallel to FMT treatment due to clinical reasons, no – broad-spectrum antibiotics could be withheld after FMT commencement and patients were off antibiotics; best discriminatory cutoffs (patients off antibiotics for >39% of days hospitalized after the first FMT) were calculated *via* receiver operating characteristics (area under the curve 0.875) using Youden’s index (0.833).

### Response to FMT and survival

**Figure 1.**
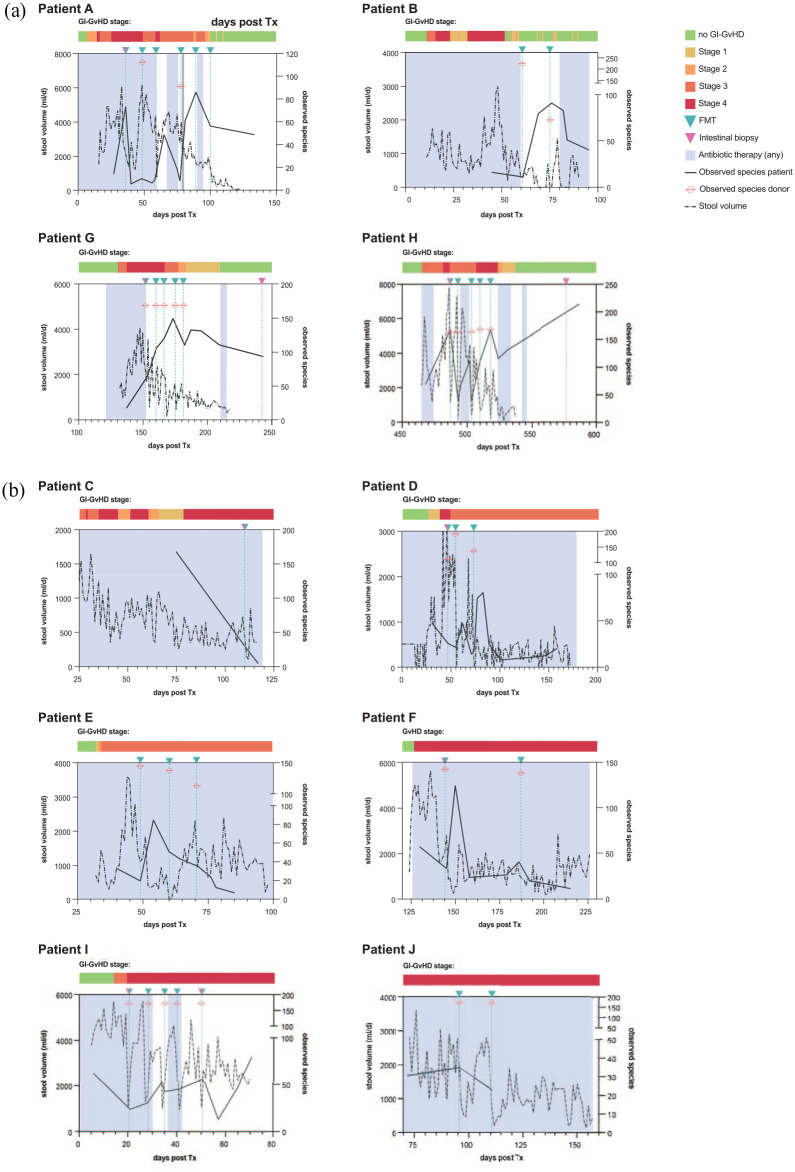
Clinical course of treatment-refractory GI-GvHD patients, split into four responders (a) and all other patients (five non-responders to FMT and patient C, panel b). Graphs show the following information over time with day 0 as the day of stem cell transplantation: GI-GvHD stage (colored bar), periods of any antibiotic use (gray background), stool volume in milliliters per day (dashed black line, left *y*-scale), time points of FMT administrations (turquoise triangles and vertical dashed lines), and time points of obtaining intestinal biopsies (pink triangles and vertical dashed lines). α-diversity of the intestinal microbiota (observed species, right *y*-scale) is shown for patients (continuous black line) and donors (crossed red circle). FMT, fecal microbiota transplantation; GI-GvHD, gastrointestinal graft-*versus*-host disease.

**Figure 2.**
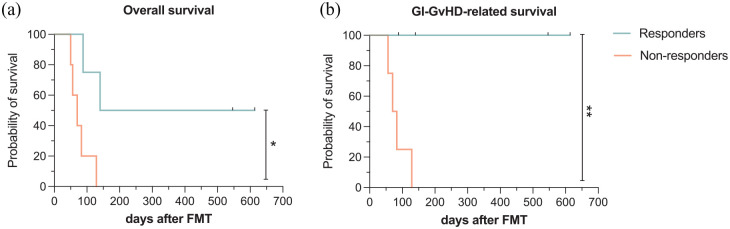
Overall survival (a) and GI-GvHD-related survival (b) stratified by response to FMT in treatment-refractory GI-GvHD. (a) Overall survival and (b) GI-GvHD-related survival after commencement of the first FMT were significantly longer in four responders (blue) *versus* five non-responders (red). FMT, fecal microbiota transplantation; GI-GvHD, gastrointestinal graft-*versus*-host disease. *p < 0.05, **p < 0.01, Log-rank test.

Sustained remission of GI-GvHD 90 days after FMT commencement (primary endpoint) was achieved in four out of nine patients who were observed longer than 14 days after the first FMT and thus included into the response analysis (i.e. responders). Patient C was not included into the response analysis due to a survival of only nine days after the first FMT. The time from the first FMT to remission of GI-GvHD was 48 days at median (6–64, remission at day 14 after first FMT in one patient). Graphs of the clinical development of all patients are compiled in Figure 1 (responders in Figure 1(a), all other patients in Figure 1(b)). Median overall survival after the first FMT was 86 days (range: 9–614, 90-day survival: 40%, *n* = 10). It was significantly longer in responders (*n* = 4, median: 140 days, range: 88–614, 90-day survival: 75%) *versus* non-responders (n = 5, median: 70 days, range: 50–129, 90-day survival: 20%) (Figure 2(a), log-rank, *p* = 0.017). In total, eight patients died during the follow-up period. Four of five non-responders, while none of the responders, died due to GI-GvHD (Figure 2(b), log-rank, *p* = 0.009). Survival plots including patient C, whose response could not be classified, are compiled in Supplementary Figure 3.

### Therapeutic differences and antibiotic use

Responders (*n* = 4) were not different to the other patients (*n* = 6) in terms of their baseline characteristics, including GvHD and GvHD therapies given concomitantly with FMTs (Table 1).

All patients received antibiotic prophylaxis with levofloxacin after allo-HSCT and prior to GI-GvHD commencement according to the local standard.

In five patients, including all four responders, FMTs could be performed partly without concomitant broad-spectrum antibiotics (Figure 1(a) and Supplementary Figure 2) because there were no proven or suspected infections at the time of FMT. In the remaining five patients, all FMTs were performed during continued prophylactic or therapeutic antibiotic administration (see Figure 1(b) and Supplementary Figure 2). Of note, none of the responders needed long-term broad-spectrum antibiotic therapy in the course of their further treatment (Figure 1(a), Table 1 and Supplementary Figure 2).

From the commencement of FMTs until the patient’s death or discharge, responders were without antibiotics for a median of 61% (equivalent to 42 days, range = 19–56 days) of the time they were hospitalized *versus* only 5% (equivalent to 1 day, range = 0–42 days) in all other patients. Receiver operating characteristics showed a good discrimination performance for the time hospitalized without antibiotics to confirm outcome (area under the curve = 0.875). The optimal cutoff used to discriminate outcome, determined by Youden’s index (0.833), was 39% of the time hospitalized without antibiotics. Using this cutoff, concomitant antibiotic administration was identified as the only therapeutic factor that could significantly discriminate responders from other patients (*p* = 0.048, Table 1).

An analysis of the use of anti-anaerobic antibiotics is separately reported in the Supplementary Results.

On average, responders received a median of five FMTs, while all other patients received a median of three FMTs (*p* = 0.510). This numerically lower number of FMTs was due to their deteriorating clinical condition owing to ongoing severe GI-GvHD and the resulting unfitness to endoscopy. Stool samples from six different FMT donors were utilized throughout the whole study, owing to the donor availability throughout the study period. However, the α-diversity of the donor microbiome administered by FMT (Figure 1) was comparable between both outcome groups (*p* = 0.540).

Second-line therapies that were given in parallel to FMTs (Table 1) were not significantly different, but trends for responders to have received less second-line therapies in addition to FMT (responders, R: *n* = 4 *versus* non-responders, NR/other: *n* = 6, *p* = 0.067) and ruxolitinib (R: *n* = 0 of 4, NR/other: *n* = 4 of 6, *p* = 0.076) were observed.

### Safety of FMT

No immediate procedure-related complications occurred during the 34 endoscopies to administer FMTs. All infectious serious adverse events that occurred after the commencement of the FMTs are summarized in Supplementary Table 1. During a 7-day period after FMTs, we observed 10 infectious events, all but one judged to be unrelated to FMTs: three cases of urinary tract infections, one case of pneumonia with *Metapneumovirus*, one case of gastroenteritis positive for *Cytomegalovirus* [polymerase chain reaction (PCR) negative in donor stool], and five cases of viremia. Of those, there was one *Polyoma BK* viremia in a patient with dysuria and high levels of *Polyoma BK virus* in urine, one reactivation of *Cytomegalovirus* (PCR negative in donor stool) needing antiviral therapy, two cases of asymptomatic and self-limiting *Adenovirus* viremia not needing specific therapy (PCR negative in donor stool), and one patient with symptomatic *Adenovirus* viremia. The latter patient received three FMTs from his asymptomatic, apparently healthy father. Four days after the third FMT (day 75), the patient, who did not respond to FMT treatment, developed multiorgan failure that was induced by an *Adenovirus* infection and died despite receiving the maximal medical support for the following 24 days. The route of infection remains unclear. Fecal transmission, endogenous reactivation, and aerogenic transmission are possible explanations. Retrospectively performed PCR of the donor stools (that were, at this time point, not yet screened prospectively for the presence of *Adenovirus*) detected *Adenovirus* in the transplant used for FMT 3, whereas earlier samples were repeatedly negative.

### Microbiota results

**Figure 3.**
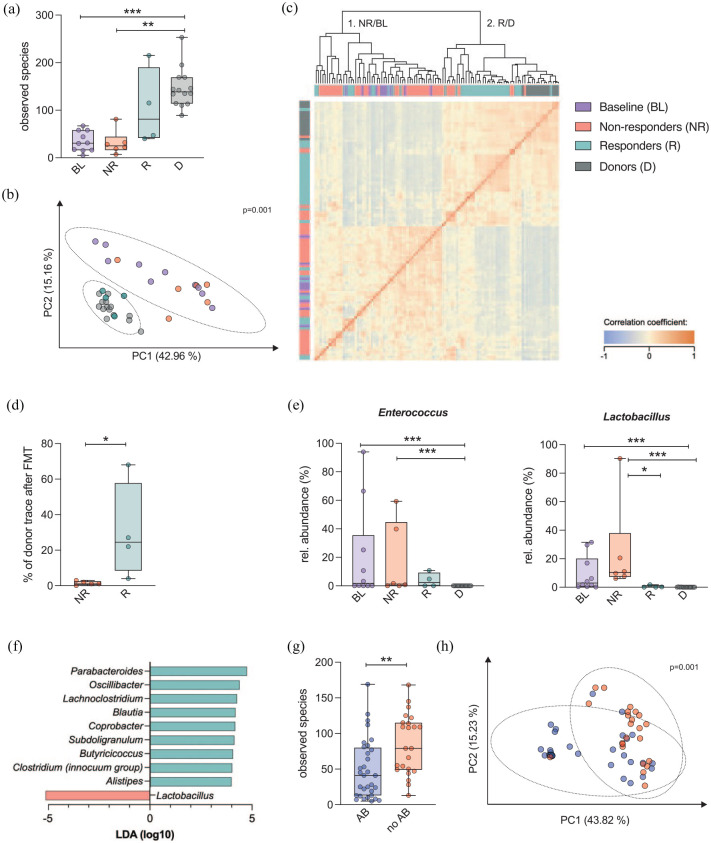
Microbiota in donors and treatment-refractory GI-GvHD patients before and after FMTs stratified according to FMT response and concomitant antibiotic use. Microbiota analyses of the four main study groups: baseline samples of all patients (BL), responders after FMT (R), all other patients (including non-responders after FMT and patient C, NR), and donors (D). (a) α-diversity (observed species), (b) β-diversity (weighted UniFrac, Principal coordinates analysis [PCoA]), (c) Spearman’s rank-order correlation of the microbiota on genus level of all samples collected, (d) proportions of microbial contents transferred from donors to non-responders as compared with responders determined by source-tracking analyses, (e) relative abundances of *Enterococcus* and *Lactobacillus*, (f) least discriminative feature analysis between responders and non-responders using LEfSe, (g) α-diversity (observed species) for samples taken after (AB) and without (noAB) antibiotic use 7 days before sampling, and (h) β-diversity (weighted UniFrac, PCoA) of samples taken during antibiotic use *versus* antibiotic withdrawal 7 days before sampling. AB, samples taken after antibiotic use 7 days before sampling; BL, baseline; FMT, fecal microbiota transplantation; GI-GvHD, gastrointestinal graft-*versus*-host disease; LEfSe, Linear discriminant analysis Effect Size; noAB, samples without antibiotic use 7 days before sampling; NR, non-responders; PCoA, Principal coordinates analysis. *p < 0.05, **p < 0.01, ***p < 0.001, Kruskal–Wallis rank sum test, Wilcoxon’s test. False discovery rate correction for multiple testing.

A significant and profound overabundance (LEfSe, Linear discriminant analysis [LDA] ⩾ 4, *p* < 0.005) of particular taxa was seen in patients at baseline (Supplementary Table 2), accompanied by a significantly lower α-diversity (observed species, *p* < 0.001) as compared with donors. The α-diversity of later responders did not differ from all other patients at baseline (*p* = 0.520). In responders, α-diversity after the last FMT (interval: 19–124 days) was comparable with that of donors (*p* = 0.291), whereas the non-responders’ α-diversity remained unchanged (*p* = 0.914, Figure 3(a)).

Regarding β-diversity, responders and donors revealed a high compositional homogeneity, whereas baseline and non-responder samples formed a more variable cluster. This was due to the frequent dominance of particular taxa in active GI-GvHD and non-responders (PCoA weighted UniFrac, PERMANOVA, *p* = 0.001, Figure 3(b)).

Spearman’s rank-order correlation of the microbiomes (genus level) showed significant positive correlations only between donors and responders. Clustering of the correlation profiles resulted in a baseline/non-responder cluster in contrast to a donor/responder cluster (Figure 3(c)). These findings again indicate the dichotomy of the responder and donor specimens, on one hand, and the non-responder and baseline microbiota profiles, on the other hand.

Source-tracking analyses revealed a transfer of 33% of the donor microbiota [mean, standard deviation (SD) 29%] to responders *versus* the transfer of 1.4% (SD 1%) to non-responders (Figure 3(d), *p* = 0.019).

The relative abundances of *Enterococcus* and *Lactobacillus* were significantly higher at baseline and in non-responders as compared with donors and responders (Figure 3(e)). Results of a discriminatory feature analysis confirmed an overabundance of *Lactobacillus* in non-responders, whereas physiological gut microbes such as *Blautia* and *Ruminococaceae*, many of which are known butyrate-producers, were found to be overabundant in responders (Figure 3(f)).

The temporary effects of antibiotic use during the 7 days preceding sampling were analyzed comparing 32 stool specimens during antibiotic administration (AB) and 23 stool specimens after a pause in antibiotic administration (noAB), irrespective of the patient’s clinical response to FMTs. NoAB samples exhibited a significantly higher α-diversity (observed species, *p* = 0.002, Figure 3(g)) as well as a clear separation from AB samples in their β-diversity (PCoA weighted UniFrac, PERMANOVA, *p* = 0.001, Figure 3(h)).

### T-cell subsets and ILC3 in GI-GvHD mucosa

**Figure 4.**
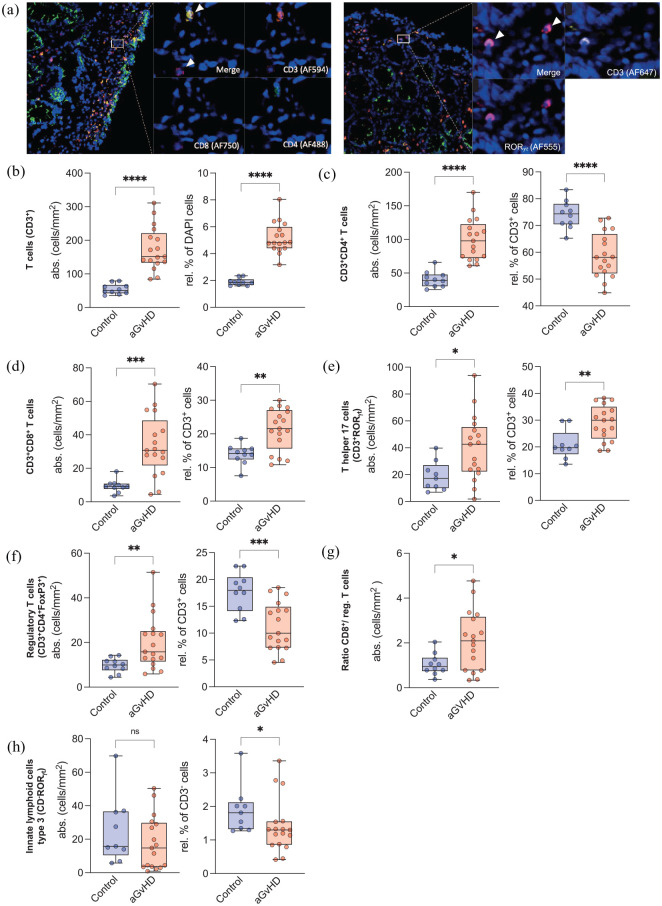
Immune cell subsets in the ileocolonic mucosa of GI-GvHD patients and healthy controls. (a) Representative staining images of CD4^+^ and CD8^+^ T cells (CD4^+^, CD3^+^/CD4^+^ and CD8^+^, CD3^+^/CD8^+^) on the left as well as innate lymphoid cells type 3 and T helper 17 cells (ILC3, CD3^–^/ROR_γT_^+^ and Th17 cell, CD3^+^/ROR_γT_^+^) to the right. White arrows denote positive cells. Quantification of absolute cell numbers (cells/mm^2^; left) and relative cell numbers (% of DAPI-, CD3^+^ cells, of patients (GI-GvHD), respectively; right) of (b) T cells – CD3^+^, (c) CD3^+^/CD4^+^ T cells, (d) CD3^+^/CD8^+^ T cells, (e) T helper 17 cells – CD3^+^/ROR_γT_^+^, (f) regulatory T cells – CD3^+^/CD4^+^/FoxP3^+^, (g) ratio of CD3^+^/CD8^+^/regulatory T cells, and (h) ILC3 cells – CD3^–^/ROR_γT_^+^ in healthy controls *versus* GI-GvHD patients. Intestinal biopsies (*n* = 25) were obtained from nine GI-GvHD patients before (*n* = 14) and after (*n* = 11) FMT and compared with biopsies (*n* = 18) from healthy mucosa taken from screening colonoscopies. DAPI, 4′,6-Diamidin-2-phenylindol; FMT, fecal microbiota transplantation; GI-GvHD, gastrointestinal graft-*versus*-host disease; ILC3, type 3 innate lymphoid cells. Mean ± SD, *p < 0.05, **p < 0.01, ***p < 0.001, ****p < 0.0001, unpaired *t*-test or Mann–Whitney test.

Immunofluorescence stainings were performed of lower intestinal biopsies obtained before the first (baseline, *n* = 14 biopsies from nine patients) and after the last fecal transfer (follow-up, *n* = 11 from four responders and four non-responders) and compared with healthy controls (*n* = 18 biopsies from three individuals). At baseline, all biopsies were taken during steroid administration and all but one patient received antibiotics (Supplementary Figure 2). Biopsy location left the distribution of immune cell subsets unaffected (Supplementary Table 3 and Supplementary Figure 6). Absolute and relative numbers of T cells were increased in GI-GvHD as compared with healthy controls (Figure 4(b), *p* < 0.001). The intestinal mucosa of patients was predominantly infiltrated by CD8^+^ T cells and Th17 cells (Figure 4(d) and (e)), while the relative numbers of CD4^+^ T cells as well as T-regs were significantly decreased (Figure 4(c) and (f)). The shift toward a more pro-inflammatory cell phenotype was also reflected by an elevated CD8^+^/T-reg ratio in patients (Figure 4(g), *p* = 0.024). Similar to T-regs, relative ILC3 levels were significantly decreased in GI-GvHD tissue (Figure 4(h), *p* = 0.045).

### Immune cell signature is associated with response to FMT

**Figure 5.**
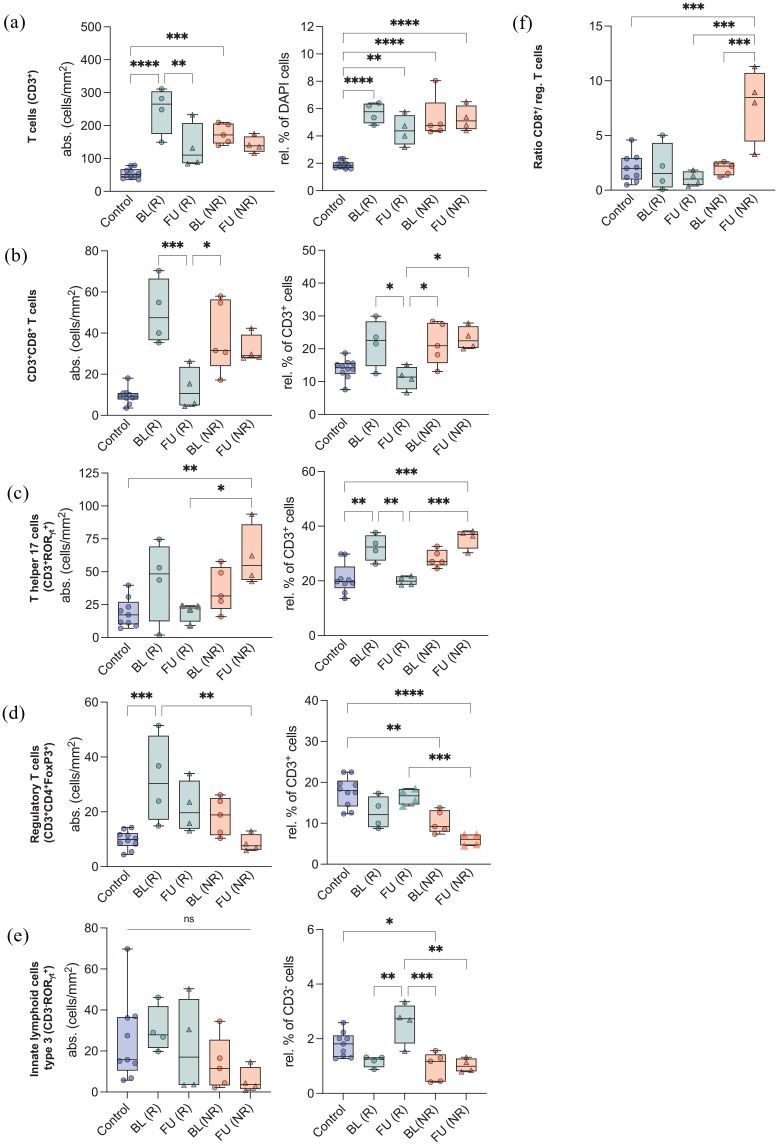
Changes of ileocolonic mucosal immune cells following FMT in GI-GvHD patients. Absolute (cells/mm^2^; left) and relative (%; right) cell numbers are depicted from healthy controls, baseline (BL) and follow-up (FU) biopsies in responders (R) *versus* non-responders (NR). (a) T cells – CD3^+^, (b) CD3^+^/CD8^+^ T cells, (c) T helper 17 cells – CD3^+^/ROR_γT_^+^, (d) regulatory T cells – CD3^+^/CD4^+^/FoxP3^+^, (e) ILC3 – CD3^–^/ROR_γT_^+^, and (f) ratio of CD3^+^/CD8^+^/regulatory T cells. Out of 11 follow-up biopsies, 5 were obtained from responders (*n* = 4), 6 were obtained from non-responders (*n* = 4), and compared with biopsies (*n* = 18) from healthy mucosa taken from screening colonoscopies. ANOVA, analysis of variance; BL, baseline; FMT, fecal microbiota transplantation; FU, follow-up; GI-GvHD, gastrointestinal graft-*versus*-host disease; ILC3, type 3 innate lymphoid cells; NR, non-responders; R, responders. Mean ± SD and one-way ANOVA and Bonferroni’s post hoc test as well as Kruskal–Wallis and Dunn’s post hoc tests. *p < 0.05, **p < 0.01, ***p < 0.001, ****p < 0.0001.

Before FMT, no significant differences in immune cell numbers between responders and non-responders were observed (Figure 5(a)–(e)). However, in patients responding to FMT, the relative numbers of CD8^+^ T cells and Th17 cells dropped after FMT (Figure 5(b) and (c), right graphic, *p* = 0.036 and 0.008) and were comparable with healthy mucosa. In contrast, CD8^+^ T cells and Th17 cells in non-responders increased after FMT and were significantly higher as compared with responders (Figure 5(b) and (c), right graphic, *p* = 0.012 and <0.001). These kinetics were inversely mirrored by T-regs, which increased in responders but dropped significantly in non-responders following FMT (Figure 5(d), right graphic, *p* < 0.001). These observations were supported by the fact that the CD8^+^/T-reg-ratio (Figure 5(f), *p* < 0.001) and Th17/T-reg ratio (*p* < 0.001) were significantly lower in responders than non-responders at follow-up. Similarly, ILC3s were restored after FMT in responders to levels seen in healthy tissue and were significantly higher to those seen in non-responders after FMT (Figure 5(e), right graphic, *p* = 0.001).

## Discussion

In this study, patients were treated with repeated colonoscopic FMTs to ameliorate severe and treatment-refractory GI-GvHD. Upon study inclusion, these patients had already received five immunosuppressive therapies on average and faced a tremendously high risk of mortality.^26^ However, we report the sustained remission of GI-GvHD in 4 out of 10 patients treated with FMT. Only patients who did not need long-term broad-spectrum antibiotic treatment responded to FMT and only those who responded to FMT were still alive 6 months after their first FMT. These results indicate that this response provides a significant survival benefit despite the small number of patients included. In a cohort of patients with exclusively severe and treatment-refractory GI-GvHD, the findings confirm previous observations of an improved survival in responders to FMT as compared with non-responders.^14–16^

Preclinical data and observations in GI-GvHD patients suggest that a severe intestinal dysbiosis accompanied by the loss of butyrate-producing anaerobes lead to intestinal short-chain fatty acid depletion and promote GI-GvHD *via* the loss of the intestinal immune system’s regulatory properties.^9,27,28^ Efforts to ameliorate dysbiosis *via* donor stool transfer by FMT led to the restoration of a more diverse microbiome and were associated with a response of GI-GvHD in a former study.^15^ However, providing antibiotic therapy concomitantly to FMTs may act as a drawback to such efforts, prolonging the dysbiosis.

In our study, microbiome engraftment was successful and four of five patients responded to FMT when antibiotics could be paused to a considerable extent before and after FMT. In five patients without an antibiotic discontinuation before or after FMTs, however, the transplanted microbiome failed to engraft, the microbial α-diversity remained low, and the patients did not respond to FMTs, even after repeated interventions. Moreover, microbiota analyses clearly demonstrated the overabundance of anaerobic butyrate-producers in responders to FMT. This result is particularly important, as the only significant clinical difference between FMT responders and non-responders was the intensity of antibiotic use. To strengthen this observation, we examined, whether the administration of antibiotics had short-term effects on the composition of the intestinal flora, an aspect that had not been addressed in former studies. Assessment of the initially greatly dysbiotic microbiota with frequent stool sampling before and longitudinally after FMTs revealed a close relationship between antibiotic use and short-term persistence of microbiota dysbiosis. Hence, antibiotic therapy is not only associated with a lack of clinical response to FMT, but antibiotic administration also seems to prevent the reconstitution of a diverse and butyrate-producing, intestinal microbiome that might be necessary for remission of GI-GvHD. In recent studies, antibiotic therapy was associated with a decline in microbiota diversity following allo-HSCT. The administration of broad-spectrum antibiotics – and especially carbapenems and piperacillin–tazobactam – which compromise anaerobic bacteria was associated with an increased GI-GvHD-related mortality.^9,27^ In contrast, a reduction in mortality was associated with the use of microbiome-preserving antibiotic regimens in allo-HSCT.^28^ Since the therapeutic administration of antibiotics is frequently necessary in GI-GvHD patients, a potential future strategy for an antibiotic regimen in patients receiving FMT for GI-GvHD needs to be discussed. Regarding this, prophylactic antibiotics and the therapeutic use of antibiotics that compromise anaerobic bacteria may be avoided whenever clinically justifiable. Whether patients with refractory GI-GvHD, who need to receive anti-anaerobic antibiotics on clinical grounds, may be unsuitable for FMT as a therapeutic option has to be clarified by future studies.

The alterations in the intestinal microbiota observed in this study show a clear dichotomy between patients at baseline and non-responders to FMT, on one hand, and healthy donors and responders, on the other hand. An initially vastly altered microbiome in terms of microbial richness and diversity during active GI-GvHD, which was dominated by taxa that are underrepresented in the physiologic gastrointestinal flora, was only replaced by a donor-like microbiota in responders. Moreover, source-tracking results suggest that the observed changes are representative of a considerable introduction of donor strains after therapy. In contrast, the non-responders’ microbiota persisted essentially unchanged without any evidence of the introduction of donor material despite repeated FMTs. The analysis of the discriminant microbial components between responders and non-responders revealed a clear picture: Genera that were overrepresented in responders were exclusively anaerobes, and virtually all of them were butyrate-producers.^29^ Moreover, strains of *Clostridia* that are known to induce T-regs in humans are among the overrepresented genera in responders.^30,31^ These observations support the role of an environment rich in butyrate, which may exert a regulatory effect on colonic epithelium in GI-GvHD, and butyrate-producers to beneficially modulate GI-GvHD.^32,33^

Still, alterations in the colonic immune cells in refractory GI-GvHD are poorly explored in humans, as most studies analyze the immune phenotype *via* peripherally circulating immune cells.^12,34^ Furthermore, human data are lacking, because the understanding of GI-GvHD has largely been driven by animal studies.^35^ In our study, we had – because of the colonoscopic application of the microbial transplant – the opportunity to obtain intestinal tissue samples immediately before FMTs and to observe changes directly in the micro-milieu of the intestinal immune system after repeated FMTs. The focus was to study the delicate balance of inflammatory and tolerogenic T-cell subsets in the intestinal mucosa in detail. Before the commencement of FMT, we observed an increase of T cells in the mucosa of patients as compared with healthy controls. In addition, pro-inflammatory cells such as CD8^+^ T cells and Th17 cells were overrepresented. The ratios of CD8^+^ and CD4^+^ T cells were significantly altered in favor of the former, which is compatible with an effector immune phenotype. Moreover, these alterations were associated with a decrease in the numbers of cells counteracting inflammatory effector cells such as regulatory T cells (T-regs) and ILC3.

The lack of balance between effector and regulatory T-cells plays an important role in the development of GvHD and ILC3 are indispensable for the homeostasis of intestinal mucosa.^34^ Studies indicate that ILC3s prevent GvHD by enhancing the regeneration of the intestinal mucosa after damage by chemotherapy.^36,37^ ILC3s reconstitute more slowly than other cell types after allo-HSCT, and higher numbers of peripherally circulating ILC3s were associated with less GvHD in humans.^38^ However, human data to ILC3 in response to GvHD-targeted therapies in affected tissues are lacking. Thus, the significant reduction in mucosal ILC3s before FMT seen in the present study may correlate immunologically with the persistent inability of the intestinal mucosa to reconstitute after the various tissue injuries evoked during allo-HSCT, resulting in GI-GvHD.

The compositional changes in the colonic immune cells after FMT in responders (i.e. the reduction in inflammation-promoting cells) suggest that an anti-inflammatory effect is induced by the therapeutic alteration of the microbiome. Accordingly, FMT has recently been shown to have an immunosuppressive effect in animal colitis models, where intestinal inflammation could be controlled *via* the induction of interleukin 10 and transforming growth factor beta, cytokines that are critical for the intestinal accumulation of T-regs.^20,21^ These findings in animal models are supported by our findings in the four treatment-refractory GI-GvHD patients who responded to FMT and showed intestinal microbiota shifts toward butyrate-producing bacterial species, which have been proven to induce T-reg activity in mice.^15,30^

The safety of an evolving, invasive treatment like the intestinal application of bacteria for GI-GvHD is of utmost importance. Substantial concerns regarding the safety were raised recently, after a patient suffered severe side effects after FMT.^39^ Several additional reports have argued the safety of FMT in immunocompromised patients after allo-HSCT.^40^ However, the iatrogenic application of fecal material *via* FMT harbors risks, both related and unrelated to the route of FMT administration. FMT application *via* a nasojejunal route is associated with the risk of regurgitation and life-threatening aspiration pneumonia.^41^ However, FMT *via* colonoscopy, as performed in this study, harbors the potential risk of colonic perforation. The potential transmission of infections *via* donor stool is inherent to both, the nasojejunal and the colonoscopic, routes of administration and remains a substantial concern regarding FMT.^39^

Recently, severe complications (sepsis, septic shock, *Norovirus* infection, bacteremia with drug-resistant bacteria) were reported as possibly or certainly related to FMT, also in GI-GvHD patients.^16,42,43^ In our study, no immediate, procedure-related complications were observed, but we reported a fatal *Adenovirus* infection in a patient who had undergone FMT. Of note, we observed a total of three patients with *Adenovirus* viremia after FMT, two of them with low viremia, asymptomatic, and self-limiting, not needing intervention. These two cases, as well as all other infectious events that occurred in vicinity to FMTs in our study, were judged to be unrelated to FMTs. However, the fatal *Adenovirus* infection may have resulted from transmission from the asymptomatic donor, who donated hematopoietic stem cells for allo-HSCT as well as stool for FMT and had frequent contact with the patient. Although *Adenovirus* reactivation can formally not be excluded, and the method of a potential transmission (i.e. *via* respiratory infection or stool) remains unclear, the possibility of an iatrogenic inoculation *via* FMT exists.

The fact that it is only possible to minimize, but not to abandon, infectious risks adherent to FMT represents a limitation of FMT for GI-GvHD and an important ethical issue to be considered in future, randomized studies that compare FMT and drug alternatives. Thus, it is compulsory to test every stool donation for enteric viral pathogens and to use frozen stool samples to allow time for testing if FMT is used in severely immunocompromised patients; such testing has been performed in our center from this time point and on. In contrast, updated recommendations for FMT donor screening still advise donor testing in 8- to 12-week intervals if donations are used repeatedly.^44^ Using this strategy, it would not have been possible to detect the *Adenovirus* in the donors’ stool in this case. We believe that specific guidelines for FMT in GvHD and other severely immunosuppressed patients including the practice of meticulously testing *every* stool donation need to be developed to ensure their safety and to address issues that arise in this special population.

This study had certain limitations. The study intervention was neither controlled nor randomized. Few randomized trials have been conducted for steroid-refractory acute GvHD, and ruxolitinib is currently the only drug approved for this indication. In a phase 3 trial, significantly higher overall response rates over standard-of-care at day 28 have been reported.^6^ However, response rates were declining at day 56 and mortality from acute GvHD did not differ significantly between the treatment arms. In contrast, in our study, response rates at day 90 post FMT seem to be sustained.

Second, the number of reported patients is small, and these results must be confirmed in a larger, multicentric study.

The observation that antibiotics may have an unfavorable effect on the FMT response cannot be causally linked, thus remains hypothesis generating, but deserves systematic confirmation. Importantly, the microbiota changes observed in responders after FMT may not be due solely to the antibiotics but also confounded by the persistent GI-GvHD in non-responders. However, antibiotics should be indicated critically, and future studies should be stratified by the type of antibiotics administered.

The changes of ileocolonic mucosal immune cells that were observed in association to FMT may be confounded by the concomitant medical therapies, especially steroids.

Finally, the administration of FMT *via* colonoscopy is demanding in treatment-refractory, severely immunosuppressed patients; thus, this technique will only be accessible in selected centers. However, it offers a unique possibility to directly obtain gastrointestinal mucosal samples and facilitate functional studies, enabling us to better understand the pathophysiological processes that lead to disease, refractoriness, and the therapeutic response in refractory GI-GvHD.

In conclusion, the results of this study fit well into the growing body of evidence pertaining to the detrimental effects of antibiotic use on clinical outcomes of allo-HSCT.^9,27^ They may serve to more clearly understand the pivotal role of antibiotic therapy in treatment-refractory GI-GvHD patients given FMT. This may allow FMTs to be more effectively adapted to patients who are likely to benefit from this intervention while avoiding a procedure with potential risks in those who have a low likelihood to respond. Future studies may implement FMT for steroid-refractory GI-GvHD as an early second-line setting, randomized to meet the standard of care. Such studies, which are – in our opinion – necessary and, given the potential of FMT in this clinical scenario, ethically justified, will only be feasible in multicentric collaborations.

The changes in the mucosal immune-cell subsets observed in treatment-refractory GI-GvHD in this study mirror findings collected under experimental conditions.^35^ They reflect the distortion of the physiological equilibrium of the intestinal immune system, shifting it toward a pro-inflammatory milieu, and the inability of the intestinal mucosa to regenerate.

Thus, a promising approach for future studies would be to support them with tissue samples from the gut to better correlate biological findings to the clinical course and to identify potential biomarkers as well as targeted therapies to treat GI-GvHD.

## Supplemental Material

sj-doc-2-tah-10.1177_20406207211058333 – Supplemental material for Antibiotic use and ileocolonic immune cells in patients receiving fecal microbiota transplantation for refractory intestinal GvHD: a prospective cohort study Click here for additional data file. Supplemental material, sj-doc-2-tah-10.1177_20406207211058333 for Antibiotic use and ileocolonic immune cells in patients receiving fecal microbiota transplantation for refractory intestinal GvHD: a prospective cohort study by Walter Spindelboeck, Bettina Halwachs, Nadine Bayer, Bianca Huber-Krassnitzer, Eduard Schulz, Barbara Uhl, Lukas Gaksch, Stefan Hatzl, Victoria Bachmayr, Lisa Kleissl, Patrizia Kump, Alexander Deutsch, Georg Stary, Hildegard Greinix, Gregor Gorkiewicz, Christoph Högenauer and Peter Neumeister in Therapeutic Advances in Hematology

sj-docx-1-tah-10.1177_20406207211058333 – Supplemental material for Antibiotic use and ileocolonic immune cells in patients receiving fecal microbiota transplantation for refractory intestinal GvHD: a prospective cohort study Click here for additional data file. Supplemental material, sj-docx-1-tah-10.1177_20406207211058333 for Antibiotic use and ileocolonic immune cells in patients receiving fecal microbiota transplantation for refractory intestinal GvHD: a prospective cohort study by Walter Spindelboeck, Bettina Halwachs, Nadine Bayer, Bianca Huber-Krassnitzer, Eduard Schulz, Barbara Uhl, Lukas Gaksch, Stefan Hatzl, Victoria Bachmayr, Lisa Kleissl, Patrizia Kump, Alexander Deutsch, Georg Stary, Hildegard Greinix, Gregor Gorkiewicz, Christoph Högenauer and Peter Neumeister in Therapeutic Advances in Hematology
