# Supplementary material for: Antibiotic use and ileocolonic immune cells in patients receiving fecal microbiota transplantation for refractory intestinal GvHD: a prospective cohort study
Source: Ther Adv Hematol. 2021 Dec 21;12:20406207211058333. doi: 10.1177/20406207211058333 (PMC8721365; doi:10.1177/20406207211058333)
Supplement: sj-doc-2-tah-10.1177_20406207211058333 – Supplemental material for Antibiotic use and ileocolonic immune cells in patients receiving fecal microbiota transplantation for refractory intestinal GvHD: a prospective cohort study [file sj-doc-2-tah-10.1177_20406207211058333.doc]

SUPPLEMENTARY METHODS

**Detailed donor stool testing for FMTs**

Potential stool donors were examined according to international FMT guidelines ^1^ for chronic and infectious diseases including their detailed patient history, physical and mental status and serological testing for hepatitis A, B and C, HIV, syphilis, *Epstein-Barr virus* and *Cytomegalovirus*. Stool was examined for the presence of occult blood by stool guaiac test, the presence of enteric pathogens (*Campylobacter*, *Salmonella*, *Shigella* and *Yersinia)* by bacterial culture, drug resistant bacteria by bacterial culture, *Clostrioides difficile* by enzyme-linked immunosorbent assay, *Noro- and Rotavirus* by PCR and the presence of intestinal parasites (including protozoa and helmiths) by microscopy. From 2017 and on, the viral screening from stool was expanded to include PCR testing for *Adenovirus, Astrovirus, Cytomegalovirus, Enterovirus, Hepatitis E-* and *Parechovirus*.
Sample storage:
Routine biopsies from the lower GI tract were placed in formaldehyde immediately after sampling. All other specimens (biopsies, stool samples) were preserved at -70°C immediately after sampling.

**Short chain fatty acid (SCFA) analyses**
We measured the SCFAs acetic acid, propionic acid, iso-butyric acid, butyric acid, iso-valeric acid, valeric acid and caproic acid extracted from stool frozen at -70 °C. Changes of SCFA between baseline and after the last FMT (delta SCFA) were compared. SCFA concentrations were measured by a 7890B/5977A MSD GC-MS (Agilent, Waldbronn, Germany) equipped with a PEG DB-WAXetr. (30m; 0,25 mm ID; 0,25 µm film) column. Stool preparation was as follows: sequential addition of 1 ml phosphoric acid (0.5 %) and 1 ml methyl-tert-butyl-ether, 10 min shaking, 10 min centrifugation and removal of the upper organic layer. 100 nmol of d-acetic acid, d-propionic acid, d-butyric acid and d-valeric acid were added as internal standards. Calibration curves by stable isotope dilution were performed from 0.1 – 2000 µM for acetic acid, propionic acid, iso-butyric acid, butyric acid, iso-valeric acid and valeric acid. Helium was used as carrier gas at 1.3 ml/min in splitless mode at 250 °C injector temperature. We maintained the initial temperature of 60 °C for 2 min and then the temperature first was ramped up to 150 °C at a rate of 15 °C/min, followed by a ramp of 5 °C/min up to 170 °C and 20 °C/min up to 250 °C where the temperature was held for 2 min. The mass spectrometer was run in electron impact (EI) mode where SCFAs were detected in SIM mode on m/z 60, 63, 73, 74, 76, 79 and 80. The source temperature was set to 250 °C and the transfer line temperature was 280 °C. Data analysis was performed by Mass Hunter (Agilent, Waldbronn, Germany).

**16S rRNA gene amplification and sequencing**Fecal microbiota composition was analyzed before FMT application (baseline), during, and after FMT treatment. Only the last sample after the last FMT was taken into account for downstream analysis of α- and β-diversity (visualization and statistics) to overcome effects of sample number bias, if not stated otherwise. The analyzed samples were categorized into donors (*n*=14), patient baseline samples (*n*=10; specimens taken before FMT), responders (*n*=4) and non-responders (*n*=6). For longitudinal analyses, all samples per patient were used.
Native fecal material was homogenized on a MagNA Lyser Instrument using MagNA Lyser GreenBeads (Roche Diagnostics GmbH, Mannheim, Germany). Bacterial DNA was extracted with the PowerLyzer PowerSoil DNA Isolation Kit (Mo Bio Laboratories, Inc., Carlsbad, CA, USA) according to the manufacturer’s instructions. Samples were processed in duplicates. Subsequently, DNA concentration was determined, and for each duplicate bacterial 16S rRNA was amplified by PCR with the Rotor-Gene SYBR Green PCR Kit (Qiagen, Hilden, Germany) using 20 ng DNA as template. To this end, the 16S primers 16s_515_S3_fwd — TGCCAGCAGCCGCGGTAA and 16s_806_S2_rev — GGACTACCAGGGTATCTAAT were used as fusion primers containing Ion Torrent sequencing adapters. To assess contaminations imported during the microbiome analysis workflow, a sample devoid of any tissue and colon content (blank) was included in the PCR run. Afterwards, PCR products were gel-purified and the amplicon DNA concentration was determined. Sequencing of pooled amplicons was performed with the Ion PGM Sequencer and an Ion Sequencing 400 Kit (both from Life Technologies, Carlsbad, CA, USA).

**Microbiota analyses**Single-end demultiplexed pyrosequencing sequences (IonTorrent) imported into QIIME2 were quality-controlled and filtered using DADA2 (denoise-pyro –p-trunc-len 276 –p-trim-lef 15)^2^. Parameters for quality-control and pre-processing are based on the reads’ quality distribution along the length of the sequence as well as on DADA2 recommendations for Ion-Torrent read data (<https://benjjneb.github.io/dada2/tutorial.html>, accessed Dec. 2019). Duplicates were pooled using mean-ceiling mode (feature-table group –p mode mean-ceiling). Subsequently, an amplicon sequencing variant (ASV) table was compiled by DADA2 based on the remaining high-quality sequencing data. For taxonomic classification of the resulting ASVs the Naïve Bayes q2-feature-classifier^3^ was trained on V4 region data (bound by primer pair 16s_515_S3_fwd TGCCAGCAGCCGCGGTAA and 16s_806_S2_rev GGACTACCAGGGTATCTAAT) extracted from the Silva reference database (version: silva_132_99_16S) ^4^. Features classified as mitochondria or chloroplasts were removed from the afore generated feature table just as features less frequent than 5 times. Default parameters were used if not specified otherwise. Further downstream statistical analysis and visualization are based on 5 287 980 (mean: 50 845.96 ± 54 473.75; min: 5 672; max: 473 855; #samples: 104) high quality sequences.
α- and β-diversity indices as well as α-rarefaction analysis (rarefication depth: 5 672) for downstream statistics and visualization was calculated by QIIME2.
Microbial composition was visualized as area charts (ASVs classified at genus level) and principal coordinate analysis (PCoA) plots (weighted UniFrac distance) ^5^ created using R (version 3.6.0) ^6^ package ggplot2 (version 3.2.1) ^7^, based on the corresponding data generated by QIIME2. Box plots representing α-diversity, relative abundance levels, and predicted donor proportions transferred, as well as graphs showing stool volumina in relation to α-diversity (observed species) and GI-GvHD grade, were also created using R (version 3.6.0) and ggplot2 (version 3.2.1).
**Multicolor immunofluorescence**After incubation with primary antibodies for 2 hours, an appropriate secondary fluorescence-labeled antibody was applied for 30 minutes at room temperature, followed by counterstaining with DAPI. Immunostainings were controlled with isotype-matched conjugates. All stainings were automatically acquired using the TissueFAXS imaging system (TissueGnostics GmbH, Vienna, Austria) installed on a Z1 Axio Observer microscope equipped with a LD Plan-Neofluar 20x/0.4 objective (Zeiss). Image analyses and quantification of cell populations were performed with the TissueQuest software (TissueGnostics GmbH, V6.01.126). Two sequential tissue sections were quantified per sample as replicates, and matched isotype controls were included. Blood vessels and intestinal glands were excluded from the analysis.

SUPPLEMENTARY RESULTS
 **Endoscopy**In total, 34 FMT applications by ileo-colonoscopy (median per patient: 3, range: 1–6) were performed. The caecum could be reached in the majority of procedures (32/34, 94%), and the terminal ileum, in 76% (26/34).
Macroscopic disease activity was most prominent in the ileum and the caecum. Ulcers were common and especially seen in the vicinity of the ileocecal valve (Supplementary Fig. 4A).
In one responder to FMT, serial imaging of the ileum showed macroscopic recovery of a completely denuded mucosa that accompanied repeated FMTs (Supplementary Fig. 4B).

**Use of anti-anaerobic antibiotics**Antibiotics that target anaerobic bacteria (Supplementary Fig. 2) were given to eight patients after FMT commencement (R: n=2 of 4, NR/other: n=6 of 6, fisher´s exact test, *p=*0.133). However, when only the use after the last FMT is considered, responders received significantly less anti-anaerobic antibiotics (R: n=0 of 4, NR/other n=5 of 6, *p=*0.048).

**Fecal short-chain fatty acids**In patients, the total amount of fecal short-chain fatty acids (SCFAs) before FMTs (mean 13 µmol/g ± SD 7) was severely decreased as compared to healthy controls (252 µmol/g ± 137, *p<*0.001, Supplementary Fig. 5). At baseline, no difference in the of total SCFAs between subsequent responders and non-responders (*p*=0.410) could be measured. The amount of SCFA increased from baseline as compared to the amount seen after the last FMT in responders (delta SCFA +14 µmol/g ± 23) as compared to the non-responders (-0.4 µmol/g ± 13). However, this difference was not significant (*p*=0.286, Supplementary Fig. 5).

SUPPLEMENTARY FIGURES

**Enrollment**

**Inclusion**

**Analysis**

Assessed for eligibility due to treatment-refractory GI-GvHD and highly reduced intestinal microbiota diversity (n=26)

Analysed (n=10)
♦ Excluded from analysis (n=0)

Included and received FMTs (n=10)

Excluded (n=16)

♦  Remission to second-line therapy other than study intervention (n=6)

♦  Unfit for endoscopy / cardiopulmonary unfit for sedation (n=9)

♦  Declined to participate (n=1)

**Supplementary Fig. 1. Patients with treatment-refractory GI-GvHD screened for FMT eligibility.**


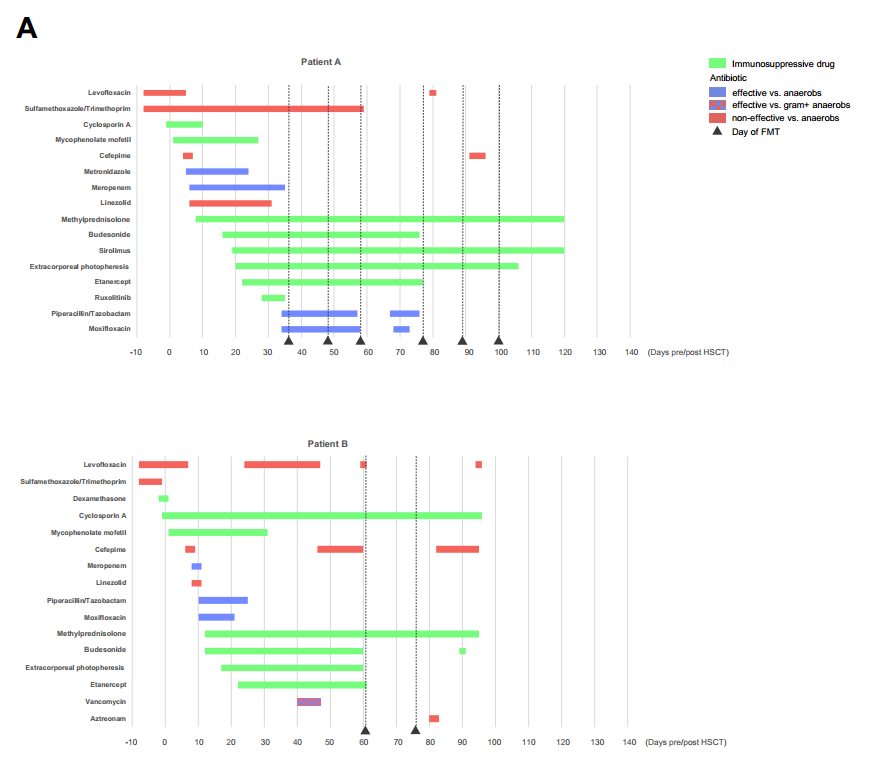

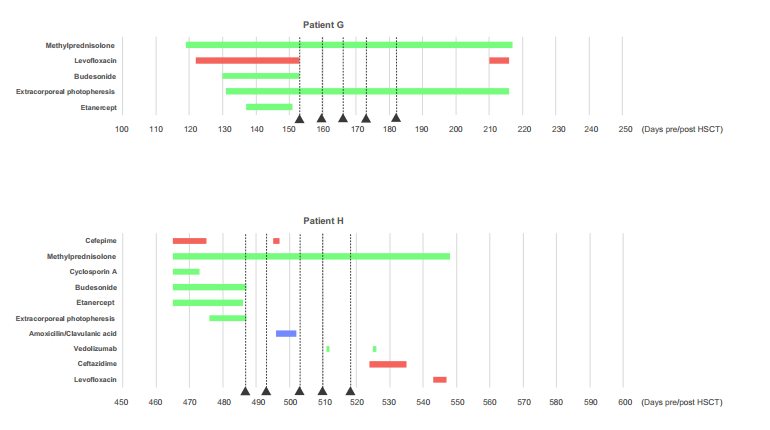


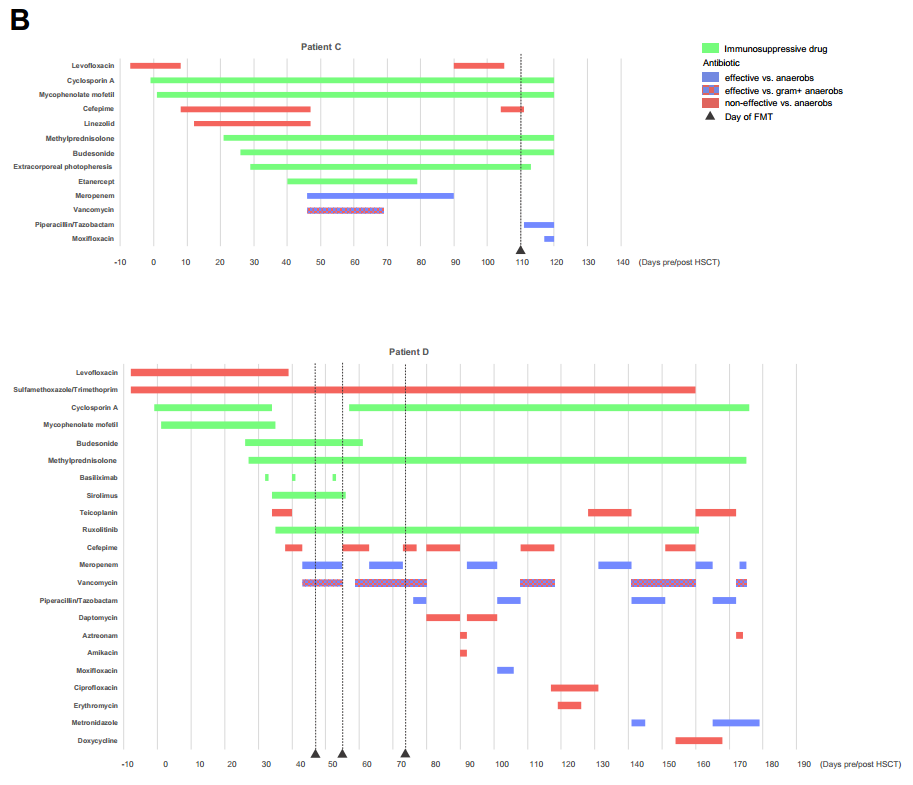

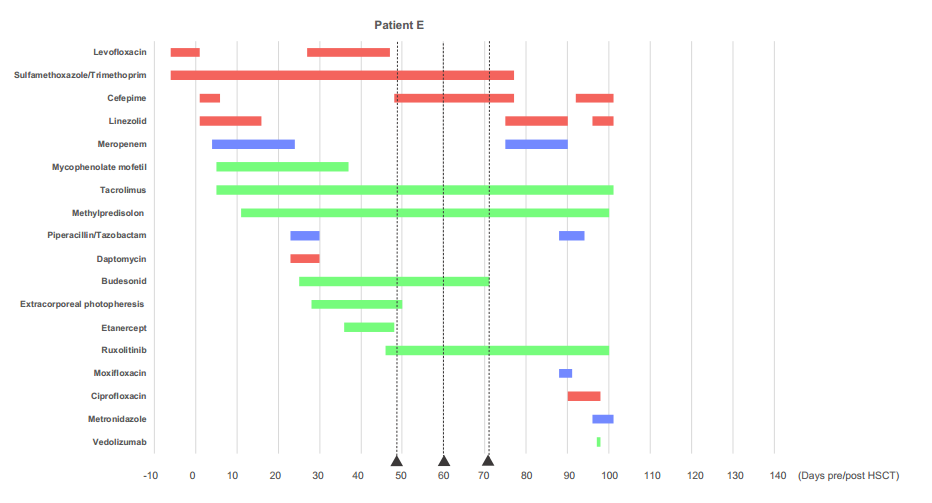


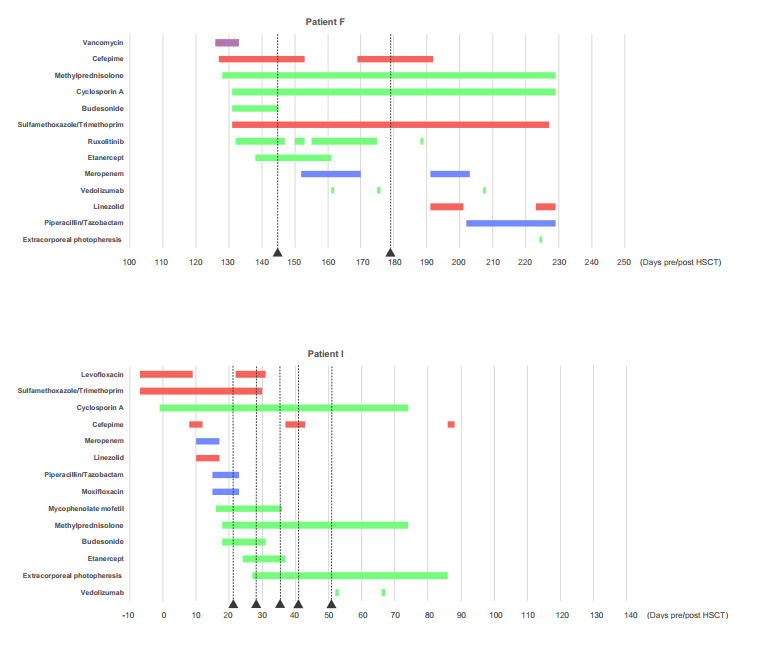

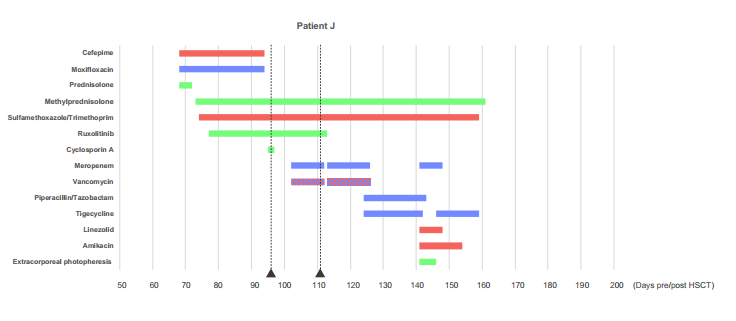


**Supplementary Fig. 2. Detailed description of antibiotics and immunosuppressants given to patients with treatment-refractory GI-GvHD.
A** Responders and **B** other patients including non-responders to FMT and patient C are displayed in relation to the days after stem cell transplantation and fecal microbiota transplantations (black triangles and dashed lines)


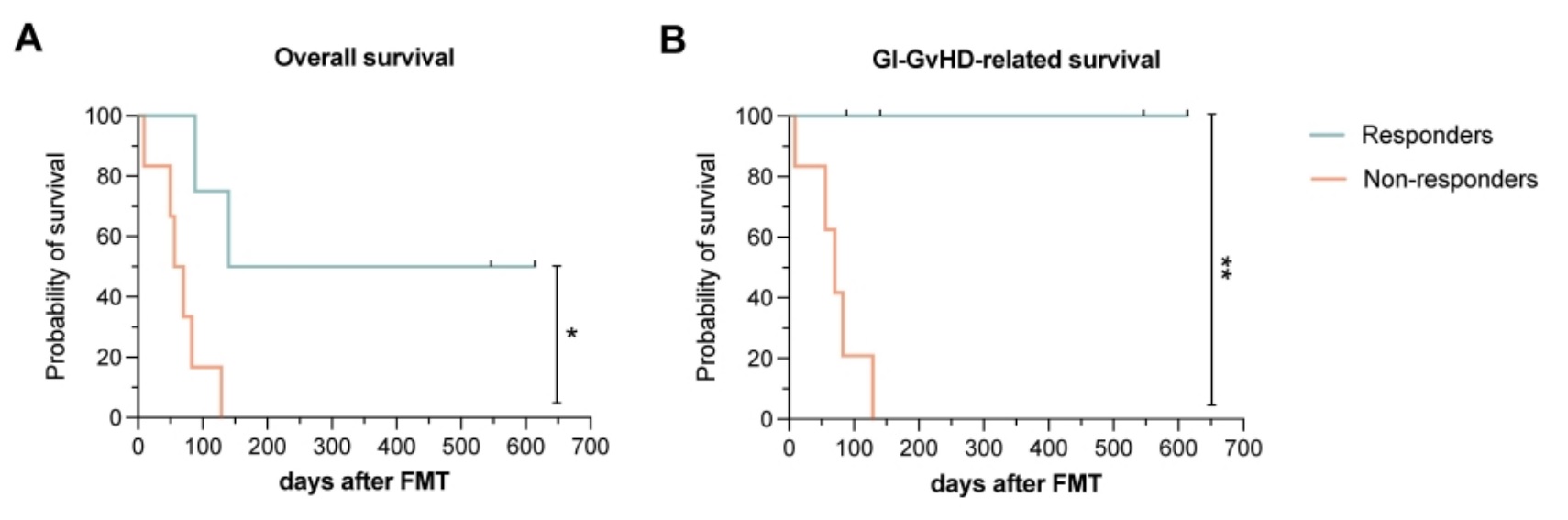


**Supplementary Fig. 3. Overall survival (A) and GI-GvHD related survival (B) stratified by response to FMT in all treatment-refractory GI-GvHD patients treated with FMT.**
(A) Overall survival and (B) GI-GvHD related survival after commencement of the first FMT were significantly longer (*p=*0.012 and *p=*0.007, respectively) in four responders (blue) versus all six other patients (non-responders and patient C, red).
***p<*0.01, **p*<0.05. Log rank test.
*GI-GvHD, gastrointestinal graft-versus-host disease; FMT, fecal microbiota transplantation*

**
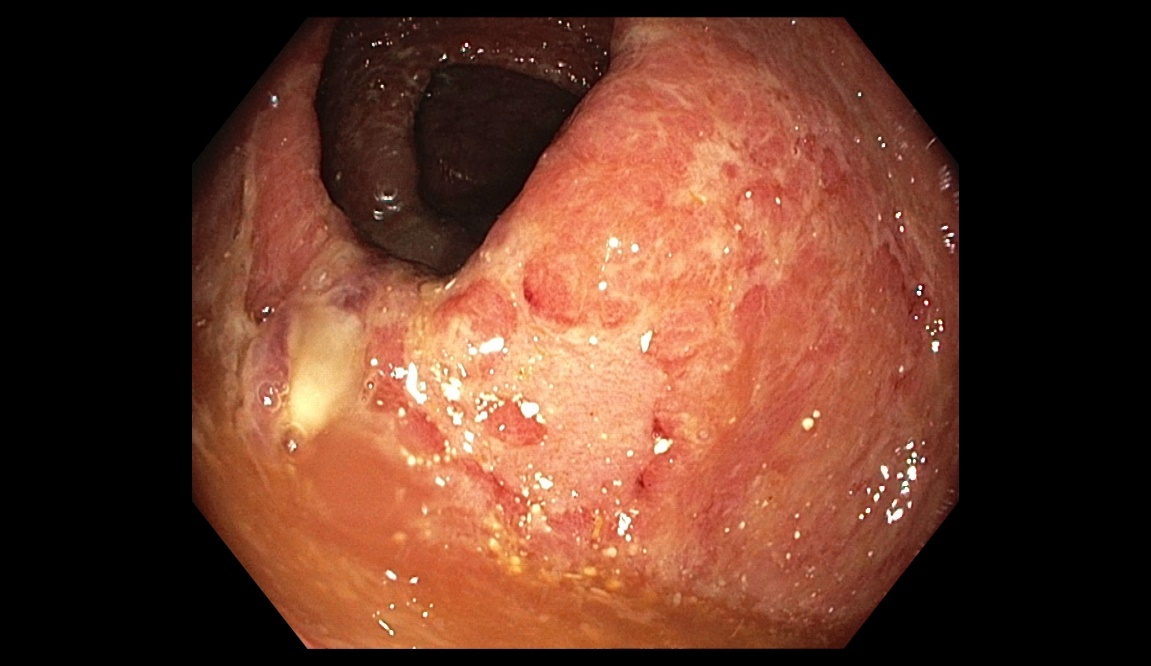


Supplementary Fig. 4. Endoscopic images of GI-GvHD.
A** Colonic ulcers: superficial ulcers, typically seen in the vincinity of the ileo-cecal valve (left and mid picture, different patients), small ulcers throughout the colon (right picture). **B** Ileitis: serial ileal imaging in a responder to fecal microbiota transplantation documents the initial complete denudation of the ileal mucosa and confluent
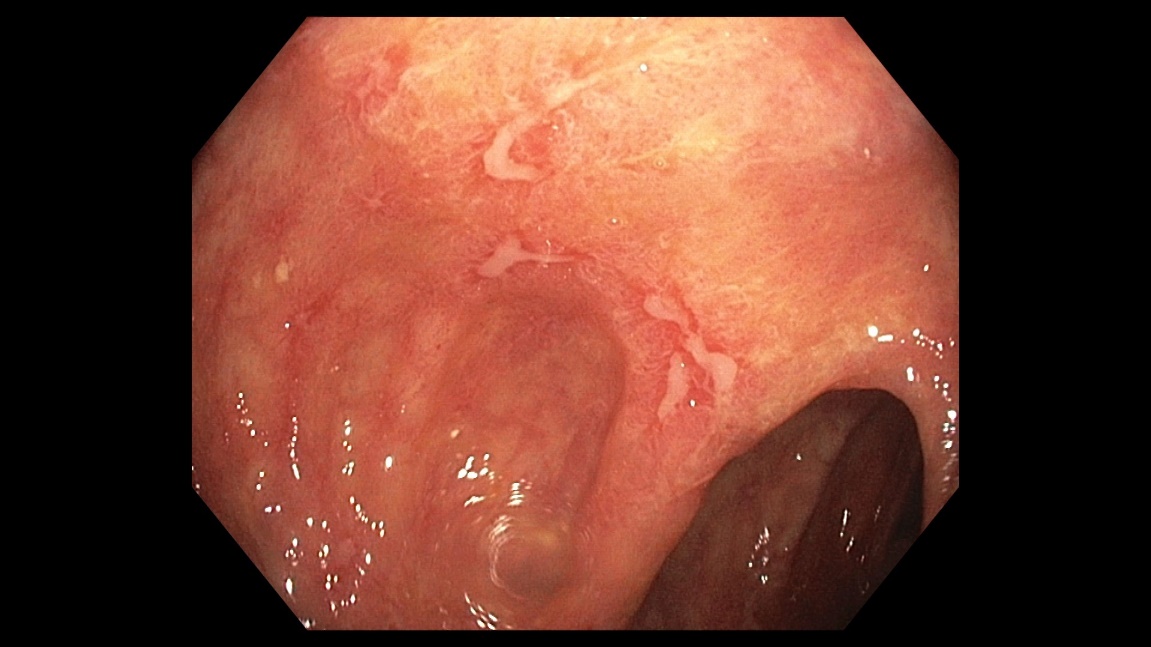

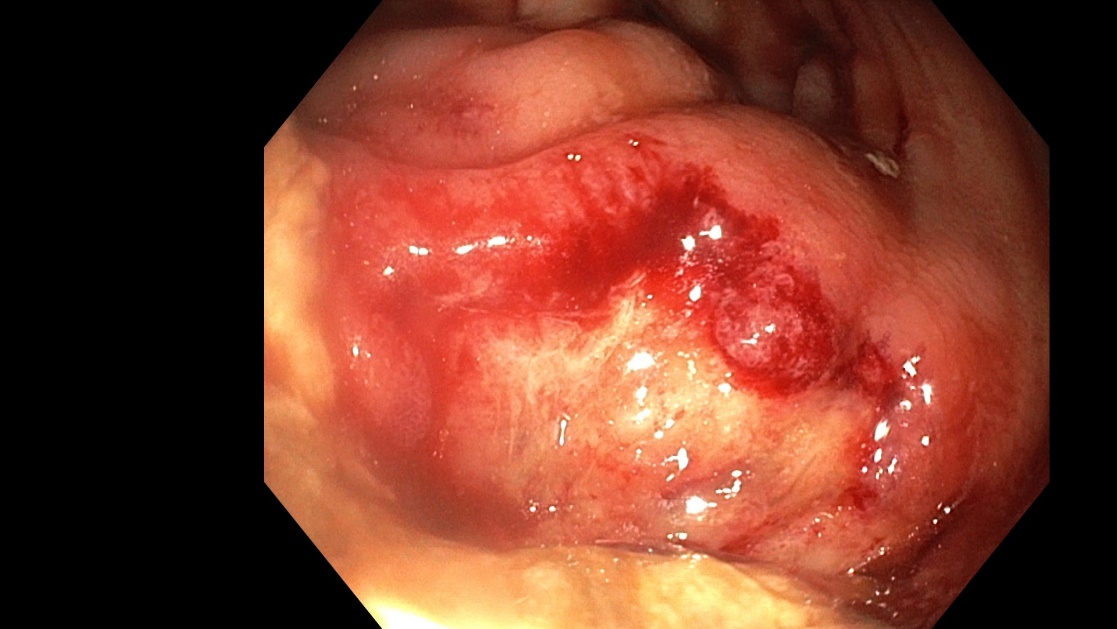

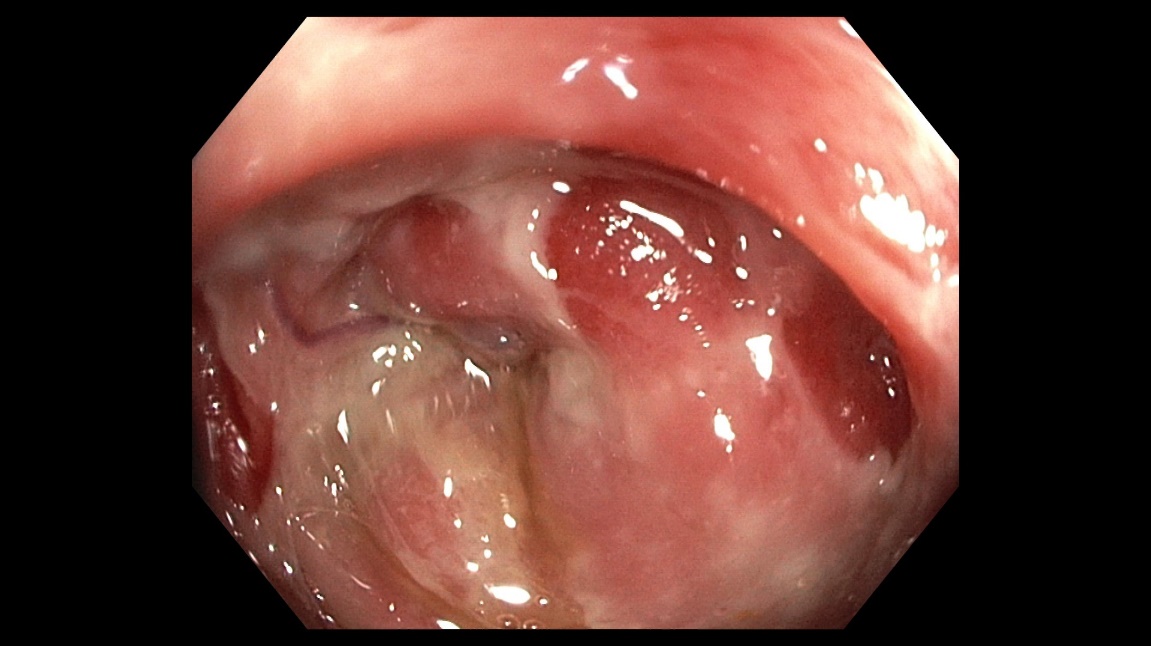

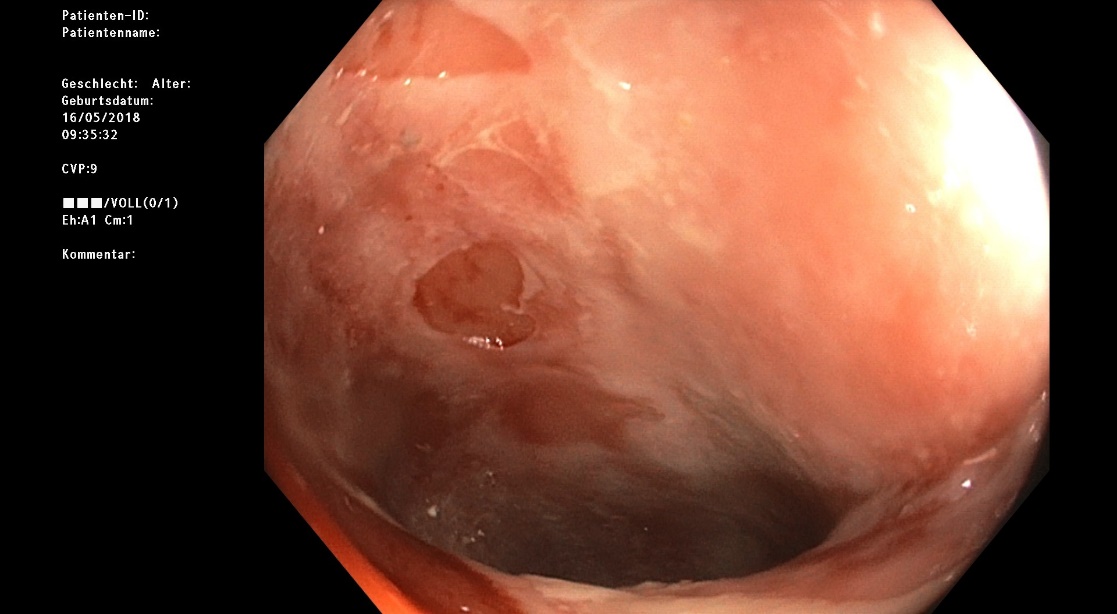

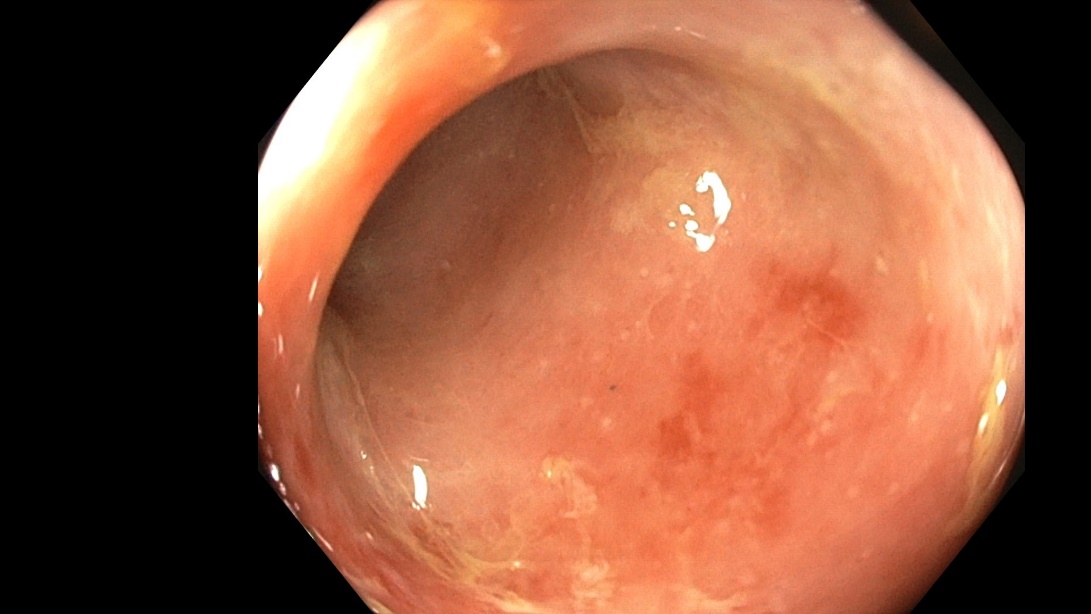
erosions (left picture) as well as stepwise healing corresponding to the reappearance of ileal mucosa (mid and right pictures; mid picture: after three FMTs and 20 days after FMT commencement; right picture: after four FMTs and 29 days after FMT commencement)

**A**

**B**

**
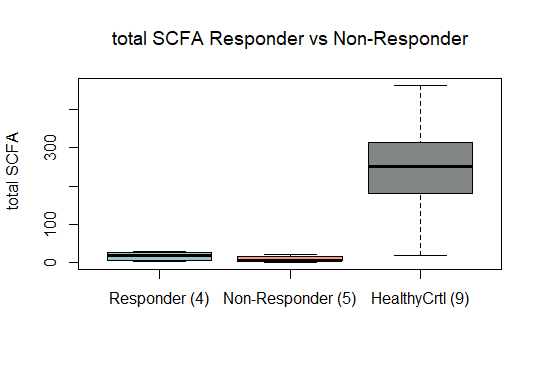
**

**A**

**
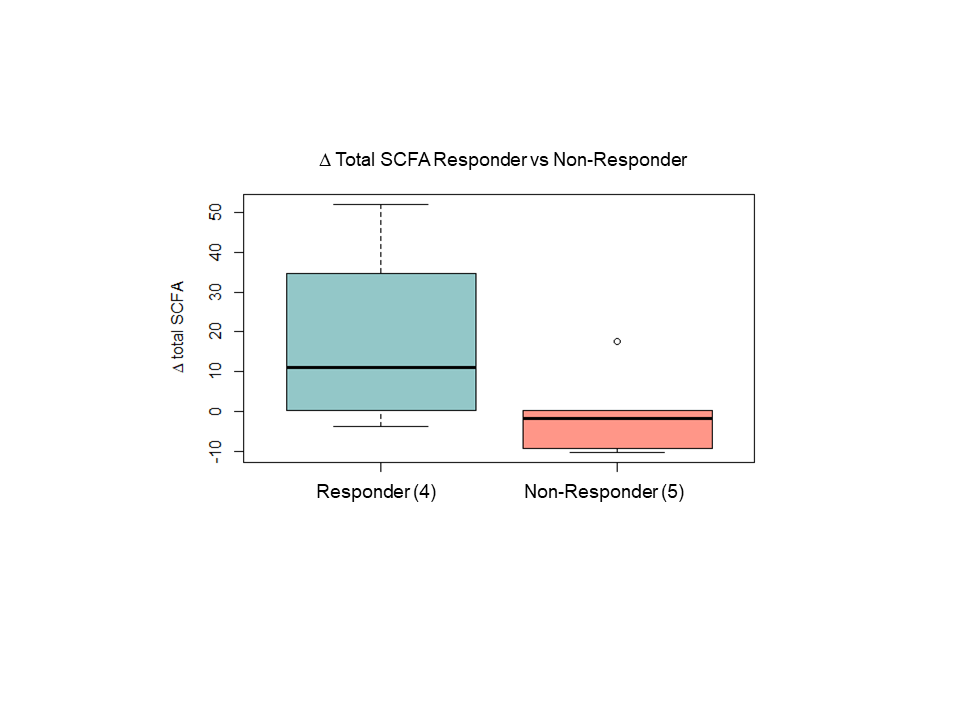
**

**B**

**Supplementary Fig. 5. Short-chain fatty acids before and after FMTs.**

**A** Short-chain fatty acid (SCFA) levels in µmol/g in stool of patients with treatment-refractory GI-GvHD at baseline as compared to healthy controls and **B** changes in SCFA levels after fecal microbiota transplantations in responders and all other patients to FMT

**Supplementary Fig. 6. Spatial distribution of lower gastrointestinal mucosal immune cells in GI-GvHD.**

**
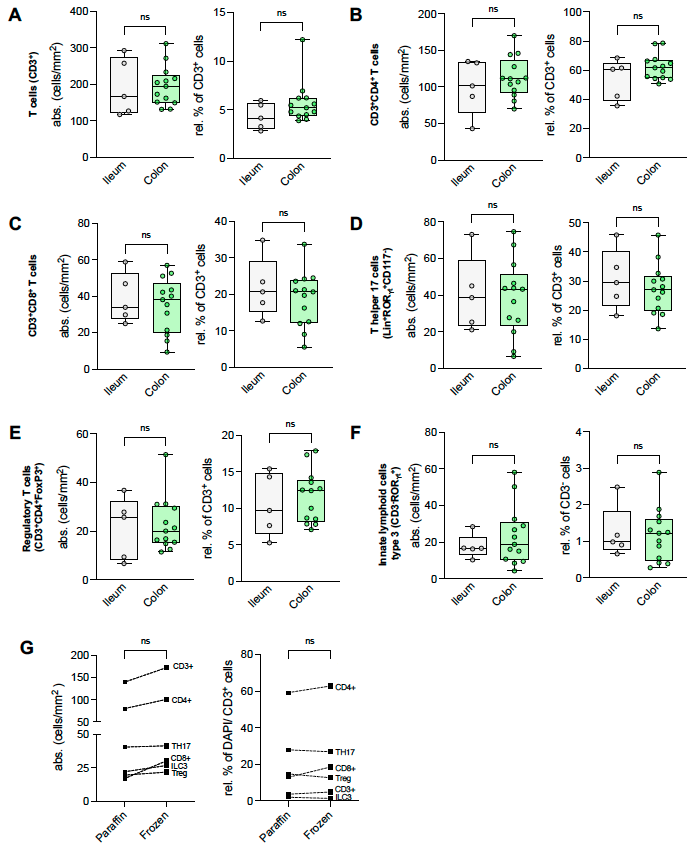
**

SUPPLEMENTARY TABLES

|  | **Patient A** | **Patient B** | **Patient C** | **Patient D** | **Patient E** | **Patient F** | **Patient G** | **Patient H** | **Patient I** | **Patient J** |
| --- | --- | --- | --- | --- | --- | --- | --- | --- | --- | --- |
| **Gender, age** | male, 53 | female, 60 | female, 61 | female, 59 | male, 24 | male, 54 | male, 42 | female, 67 | male, 39 | female, 51 |
| **Hematologic disease** | MDS | Secondary AML/ MDS | AML | AML | MDS | PMF | MDS | PMF | Multiple myeloma | AML |
| **Donor** | female URD (HLA 9/10, HLA-DQB1 mismatch) | female URD | male MSD | male URD | 1. female HID; 2.male HID | female MSD | female URD | male URD | female MRD | male URD |
| **GvHD prophylaxis*** | CsA, MMF | CsA, MMF | CsA, MMF | CsA, MMF | 1. Cyclo,CsA,MMF; 2.Cyclo,Tacrolimus,MMF | CsA, MMF | CsA, MTX | CsA, MMF | CsA, MMF | CsA, MMF |
| **Onset of GvHD (days after HSCT; organ, stage)** | +7; skin 1 | +10; skin 2 | +19; upper GI | +20; skin 1 | +25; skin 2 | +122; skin 1 | +116; liver 3 | +465; liver 2, GI 3 | +18; lower GI 3 | +71; lower GI 4, skin 2 |
| **Onset of lower GI-GvHD (days after HSCT)** | +12 | +11 | +22 | +26 | +34 | +124 | +123 | +465 | +18 | +72 |
| Diarrhea peak (days after HSCT; volume) | +47; 6200 ml | +48; 3000 ml | +31; 1700 ml | +43; 3200ml | +44; 3600ml | +137; 5100ml | +146; 4000ml | +486; 7800ml | +25; 5400ml | +86; 3050 ml |
| **GvHD therapy prior to FMT (duration in days)** |  |  |  |  |  |  |  |  |  |  |
| Budesonide | 21 | 47 | 84 | 20 | 23 | 15 | 25 | - | 21 | - |
| ECP (number of applications) | 17 (6x) | 42 (16x) | 81 (20x) | - | 21 (10x) | - | 27 (12x) | 10 (5x) | 8 (3x) | - |
| Etanercept / *Basiliximab* (number of applications) | 15 (5x) | 38 (12x) | 52 (12x) | *14 (3x)* | 13 (4x) | 5 (2x) | 16 (5x) | 18 (6x) | 11 (3x) | - |
| Methylprednisolone (MP) | 30 | 49 | 89 | 19 | 35 | 18 | 34 | 21 | 17 | 21 |
| Ruxolitinib | 7 | - | - | 12 | 4 | 14 | - | - | - | 19 |
| Sirolimus/CsA/Tacrolimus | Sirolimus 18 | - | - | Sirolimus 12 | Tacrolimus 44 | CsA 20 | - | - | - | - |
| **GvHD therapy at time of first FMT** | Budesonide, ECP, Etanercept, MP, Sirolimus | CsA, MP, Budesonide | Budesonide, CsA, ECP, MP, MMF | MP, Sirolimus, Basiliximab, Budesonide | MP, Tacrolimus, Etanercept, Ruxolitinib, Budesonide | CsA, MP, Budesonide, Etanercept, Ruxolitinib | MP, Etanercept, ECP | MP, Etanercept, ECP | MP, CsA, MMF, Etanercept, ECP | MP, Ruxolitinib |
| **Overall GvHD grade at time of first FMT ^8^** | IV | IV | IV | IV | IV | IV | IV | IV | IV | IV |
| Skin (Stage) | 0-1 | 0 | 0 | 0 | 0 | 0 | 0 | 0 | 0 | 2 |
| Liver (Stage) | 0 | 0 | 0 | 0 | 0 | 0 | 0 | 0 | 0 | 0 |
| Lower GI (Stage) | 4 | 2 | 4 | 4 | 4 | 3 | 4 | 4 | 4 | 4 |
| **GI GvHD at time of first FMT** |  |  |  |  |  |  |  |  |  |  |
| Endoscopic results | Hemorrhagic colitis, fibrinous ulcers | Ileitis hemorrhagic colitis | ulcerous ileitis, Colon: loss of normal vascular pattern, no erosions or ulcers | Ulcerous ileitis, denuded mucosa | Ulcerous pancolitis | Erosive pancolitis | Erosive gastritis, Ileitis, denuded mucosa | Ulcerous colitis | Ulcerous colitis | Ulcerous, hemorrhagic colitis |
| Histology (Lerner grade) | 3 | 1-2 | 1-2 | 4 | 3 | 1-2 | 2-3 | 1-2 | 3 (duodenum) | 3 |
| **Number of FMTs** | 6 | 2 | 1 | 3 | 3 | 2 | 5 | 5 | 5 | 2 |
| **Timepoint of first/last FMT (days after HSCT)** | +37 - +100 | +61/+76 | +110 | +46/+54/+73 | +49/+66/+71 | +143/+176 | +153 - +182 | +487 - +525 | +35 - +65 | +95/+109 |
| **GI GvHD outcome** |  |  |  |  |  |  |  |  |  |  |
| Clinical response | CR, discharge on day +119 w/o GVHD symptoms | CR, discharge on day +95 w/o GVHD symptoms | n/a (follow-up < 14 days after FMT 1) | PR, diarrhea diminished to < 500ml | NC | NC | CR, discharge on day +215 w/o GvHD symptoms | CR, discharge on day +564 w/o GvHD symptoms | NC | NC |
| Endoscopical | n/a | n/a | n/a | Ulcerous ileitis | PD with ulcera (entire colon + rectum) | GvHD p.m. terminal ileum and denuded mucosa | Ongoing ileitis, colon CR | Ulcus in ileocecal region | Erosive ileitis | Ulcerous hemorrhagic enterocolitis |
| Histological (Lerner grade) | n/a | n/a | n/a | 1 | n/a | 1-2 | 1 (improvement, no apoptosis) | 0 (no apoptosis) | 3-4 | 1 |
| **Infectious events after first FMT (days after HSCT; days after the preceding FMT; pathogen)** | *Bacteremia (+68; +10; E. coli) *UTI (+78; +1; E. coli) *UTI (+89; +12; E. coli) *CRBSI (+134; +34; E. coli) *Neutropenic sepsis (+176; +76; E. coli and Klebsiella pneumoniae) | *UTI (+80; +4; Pseudomonas species and Klebsiella pneumoniae) *Acute purulent pansinusitis with secondary meningitis (+129; +53; n/a) | *UTI (+116; +6 ; Enterococcus) *Pneumonia (+116; n/a) | *Gastroenteritis (+76; +3; CMV) | *Pneumonia, viremia, liver failure (+75; +4; Adenovirus) | *Pneumonia (+179; +3; Metapneumovirus) *Repetitive CMV-reactivations | - | *Viremia (+496; +3; Adenovirus) | *Viremia (+37; +2; Polyoma BK reactivation, +72; +7; Adenovirus) *Bacteriemia (+91; +26; Enterococcus) | *Viremia (+101; +6; CMV reactivation) *Infection with undetermined origin (+122; +13; n/a) |
| **Death (due to uncontrolled GI-GvHD yes/no; alternative cause; days after HSCT)** | no; septicemia; +177 | no; cerebral infarction; +148 | yes; n/a; +119 | yes; n/a; +173 | no; Adenovirus MOF; +99 | yes; n/a; +362 | no; n/a; alive | no; n/a; alive | yes; n/a; +91 | yes; n/a; +165 |

**Supplementary Table 1. Detailed patient characteristics.**GvHD, graft-versus-host disease; allo-HSCT, allogeneic hematopoietic stem cell transplantation; AML, acute myeloid leukemia; CR, complete remission; CRBSI, catheter-related bloodstream infection; CMV, Cytomegalie virus; CsA, cyclosporine A; Cyclo, Cyclophosphamide; E. coli, Escherichia coli; ECP, extracorporal photopheresis; FMT, fecal microbiota transplantation; GI, gastrointestinal; HID, haploidentical donor; HLA, human leukocyte antigen; MP, methylprednisone; MMF, mycophenolate mofetil; MOF, multiorgan failure; MDS, myelodysplastic syndrome; MRD, matched related donor; MSD, matched sibling donor; n/a, not applicable; NC, no change; nd, not done; PBSC, peripheral blood stem cells; PD, progressive disease; p.m., punctum maximum; PMF, primary myelofibrosis; PR, partial response; URD, unrelated donor; UTI, urinary tract infection;
* none of the patients received antithymocyte globulin.

|  |  |  | Patients pre-FMT | | | | Healthy controls (donors) | | | |
| --- | --- | --- | --- | --- | --- | --- | --- | --- | --- | --- |
| Genus | LEfSe | p | median | mean | minimum | maximum | median | mean | minimum | maximum |
|  | | | | | | | | | | |
| *Enterococcus* | 5.3 | 0.000 | 1.7 | 20.0 | 0.0 | 94.0 | 0.0 | 0.0 | 0.0 | 0.0 |
| *Lactobacillus* | 5.0 | 0.000 | 2.9 | 9.2 | 0.0 | 31.5 | 0.0 | 0.0 | 0.0 | 0.3 |
| *Campylobacter* | 4.5 | 0.001 | 0.0 | 3.3 | 0.0 | 23.6 | 0.0 | 0.0 | 0.0 | 0.0 |
| *Staphylococcus* | 4.3 | 0.004 | 0.2 | 2.2 | 0.0 | 13.2 | 0.0 | 0.0 | 0.0 | 0.0 |

**Supplementary Table 2. Linear discriminant analysis results, patients vs. healthy controls, at baseline.**FMT, fecal microbiota transplantation; LEfSe, linear discriminant analysis effect size; Kruskal-Wallis rank sum test

| **Sample** | **Location** | **Timepoint** | **FMT-response** | **Patient ID** |
| --- | --- | --- | --- | --- |
| 1 | Sigmoid colon | BL | n/a | C |
| 2 | Ascending colon | BL | n/a | C |
| 3 | Sigmoid colon | BL | later R | A |
| 4 | Sigmoid colon | BL | later NR | D |
| 5 | Ileum | BL | later NR | F |
| 6 | Caecum | BL | later NR | F |
| 7 | Ileum | BL | later R | G |
| 8 | Caecum | BL | later R | G |
| 9 | Sigmoid colon | BL | later R | H |
| 10 | Ileum | BL | later NR | I |
| 11 | Caecum | BL | later NR | I |
| 12 | Sigmoid colon | BL | later NR | I |
| 13 | Ileum | FU | R | G |
| 14 | Ascending colon | FU | R | G |
| 15 | Sigmoid colon | FU | R | H |
| 16 | Ileum | FU | NR | I |
| 17 | Ascending colon | FU | NR | I |
| 18 | Sigmoid colon | FU | NR | I |

**Supplementary Table 3A. Cohort of cryo-samples for immunofluorescence stainings indicating biopsy location, time point of sampling, response status for follow-up samples and Patient ID.**BL= baseline pre FMT, FU= follow-up post FMT; response to FMT-treatment: R=responder, NR=non-responder. If several samples were taken from a patient at a single time point a frame summarizes them.

| **Sample** | **Timepoint** | **FMT-response** | **Patient ID** |
| --- | --- | --- | --- |
| 19 | BL | later R | B |
| 20 | BL | later NR | J |
| 21 | FU | R | A |
| 22 | FU | R | B |
| 23 | FU | NR | D |
| 24 | FU | NR | E |
| 25 | FU | NR | J |

**Supplementary Table 3B. Cohort of paraffin samples for immunofluorescence stainings indicating time point of sampling, response status for follow-up samples and patient ID.**BL= baseline pre FMT, FU= follow-up post FMT; response to FMT-treatment: R=responder, NR=non-responder. If several samples were taken from a patient at a single time point a frame summarizes them.

| **Target** | **Supplier** | | **Clone** | **Lot-number** | | | **Dilution OCT** | **Dilution Paraffin** |
| --- | --- | --- | --- | --- | --- | --- | --- | --- |
| CD3 | Biolegend | | UCHTI | B270481 | | | 0,25µg/ml | 0,12µg/ml |
| CD3 | BD | | SK7 | 7170831 | | | - | 1µg/ml |
| CD4 | BD | | SK3 | 7223652 | | | 0,3µg/ml | 0,15g/ml |
| CD8 | R&D | | #37006 | AELJ0219111 | | | 2,5µg/ml | - |
| CD8 | Biolegend | SK1 | | | B120392 | - | | 0,12µg/ml |
| FoxP3 | Biolegend | | 206D | B244884 | | | 1µg/ml | 1µg/ml |
| ROR_γT_ | eBioscience | | AFKJS-9 | 2158265 | | | 4µg/ml | 4µg/ml |

**Supplementary Table 4: Source and type of antibodies.**

Supplementary References

1. Cammarota G, Ianiro G, Tilg H, et al. European consensus conference on faecal microbiota transplantation in clinical practice. Gut. 2017;66(4):569-580.

2. Callahan BJ, McMurdie PJ, Rosen MJ, Han AW, Johnson AJ, Holmes SP. DADA2: High-resolution sample inference from Illumina amplicon data. Nat Methods. 2016;13(7):581-583.

3. Bokulich NA, Kaehler BD, Rideout JR, et al. Optimizing taxonomic classification of marker-gene amplicon sequences with QIIME 2's q2-feature-classifier plugin. Microbiome. 2018;6(1):90.

4. Pruesse E, Quast C, Knittel K, et al. SILVA: a comprehensive online resource for quality checked and aligned ribosomal RNA sequence data compatible with ARB. Nucleic Acids Res. 2007;35(21):7188-7196.

5. Lozupone CA, Hamady M, Kelley ST, Knight R. Quantitative and qualitative beta diversity measures lead to different insights into factors that structure microbial communities. Appl Environ Microbiol. 2007;73(5):1576-1585.

6. Team RC. R: A language and environment for statistical computing. Vienna, Austria: R Foundation for Statistical Computing, 2013.

7. Wickham H. ggplot2: Elegant Graphics for Data Analysis. Springer International Publishing, 2016.

8. Przepiorka D, Weisdorf D, Martin P, et al. 1994 Consensus Conference on Acute GVHD Grading. Bone Marrow Transplant. 1995;15(6):825-828.
